# The Combination of Resveratrol and Conjugated Linoleic Acid Dienes Enhances the Individual Effects of These Molecules on De Novo Fatty Acid Biosynthesis in 3T3-L1 Adipocytes

**DOI:** 10.3390/ijms252413429

**Published:** 2024-12-14

**Authors:** Jarosław Oczkowicz, Ewelina Piasna-Słupecka, Mariola Drozdowska, Aneta Koronowicz, Aneta Kopeć

**Affiliations:** Department of Human Nutrition and Dietetics, University of Agriculture, Balicka 122, 30-149 Kraków, Poland

**Keywords:** resveratrol, *cis-9*, *trans-11* conjugated linoleic acid (CLA), *trans-10*, *cis-12* CLA, 3T3-L1 adipocytes, de novo fatty acid biosynthesis

## Abstract

Consuming food containing ingredients with a documented impact on lipid metabolism can help fight overweight and obesity. The simplest way to reduce the level of fatty acids is to block their synthesis or increase the rate of their degradation. This study aimed to determine the effect of resveratrol, *cis-9*, *trans-11* conjugated linoleic acid (CLA), *trans-10*, *cis-12* CLA, and various variants of their combinations on de novo fatty acid biosynthesis in 3T3-L1 adipocytes. The influence of the above-mentioned bioactive substances on cells grown under standard conditions and after induction of oxidative stress was measured. The effect of the tested compounds on the expression of selected genes related to the de novo fatty acid biosynthesis process (Fasn, Acc1, Acly, Prkaa1, Prkaa2, Prkaca, Srebp1) was evaluated. As part of the conducted experiments, how the level of the corresponding mRNA translates into the content of selected proteins (acetyl-CoA carboxylase 1 (ACC) and fatty acid synthase (FASN) was studied. It was found that the inhibition of fatty acid biosynthesis processes was stronger in the case of the combination of the tested CLA isomers (*cis-9*, *trans-11* CLA, *trans-10*, *cis-12* CLA) with resveratrol than in cases of their individual action.

## 1. Introduction

Obesity is a significant global health issue that has been impacting quality of life and increasing the risk of related diseases for decades [1]. However, with decisive action and a commitment to change, we can overcome this challenge and improve people’s health. According to the WHO’s data from 2022, over 2.5 billion adults worldwide were affected by overweight and obesity, making these a concern [1]. Improper eating habits, such as excessive consumption of products rich in digestible sugars and high in fats, are the leading causes of obesity. Triacylglycerols are a major energy source stored in cells, particularly those that make up adipose tissue and skeletal muscles, and are broken down into glycerol and fatty acids when required. Obesity is a complex trait influenced by multiple genetic and environmental factors. Genes play a significant role in determining an individual’s susceptibility to obesity by affecting various physiological processes such as appetite regulation, energy expenditure, fat storage, and metabolism. Genetic variations can influence how efficiently the body uses and stores energy, which can lead to differences in body weight and fat distribution among individuals. The key genes involved in obesity are FTO, MC4R, LEP, LEPR or PPARG [2]. For example, fat mass and obesity-associated (FTO) gene is essential for insulin secretion and beta-cell function, as indicated in in vitro studies using INS-1 cells and human pancreatic islets. FTO is a major genetic link between breast cancer, obesity, and diabetes [3,4,5] PPARG regulates adipocyte differentiation, lipid storage, and glucose metabolism, affecting insulin sensitivity and linking it to obesity, metabolic syndrome, and type 2 diabetes [6]. Melanocrtin 4 receptor (MC4R) is critical for appetite and energy balance through hypothalamic signaling, where mutations often result in increased hunger and reduced satiety, contributing to obesity [7]. Leptine (LEP) is mainly responsible for the encoding of peptides important for the energy homeostasis produced by white adipose tissue [8]. Leptin receptor (LEPR) encodes the leptin receptor, essential for leptin signaling, and mutations here contribute to leptin resistance and increased food intake [9]. Changes in the expression of genes related to fatty acid metabolism are frequently linked to metabolic syndrome and insulin resistance, which are risk factors for various diseases caused by excessive triacylglycerol levels in adipocytes, including type 2 diabetes, atherosclerosis, hyperlipidemia, and arterial hypertension [10]. Disruptions in the body’s oxidative potential, which are associated with obesity, can be effectively countered by reducing adipose tissue content. Reducing energy supply and consuming a diet rich in antioxidants, such as vitamins A, E, and C or flavonoids, is crucial in preventing obesity complications [4,11].

Obesity is characterized by increased oxidative stress in adipose tissue, which is strongly associated with the synthesis of adipokines and pro-inflammatory cytokines. These factors can contribute to the development of metabolic syndrome [12]. Reactive oxygen species (ROS) in the body have been shown to cause protein and lipid oxidation, as well as DNA damage, leading to changes in gene expression. The human body possesses its own defense mechanisms against free radicals, which include antioxidant enzymes such as glutathione peroxidase (GPX), glutathione reductase (GR), and superoxide dismutase (SOD). In cases of obesity, the activity of antioxidants is often insufficient to reduce oxidative stress. Therefore, biologically active substances are needed that can support or partially replace their action. Both plant and animal products are excellent sources of many antioxidant and health-promoting substances. These compounds protect the body against the harmful effects of free radicals, reducing the risk of oxidative stress [13,14]. Consuming food that contains ingredients with a documented impact on the body’s lipid metabolism can significantly aid in the fight against overweight and obesity. The most effective approach to reducing the level of fatty acids is to block their synthesis or increase the rate of their degradation. By reducing the transcription of genes related to the biosynthesis of fatty acids or increasing the expression of genes associated with the process of their oxidation, weight reduction can be supported [15].

**Figure 1:**
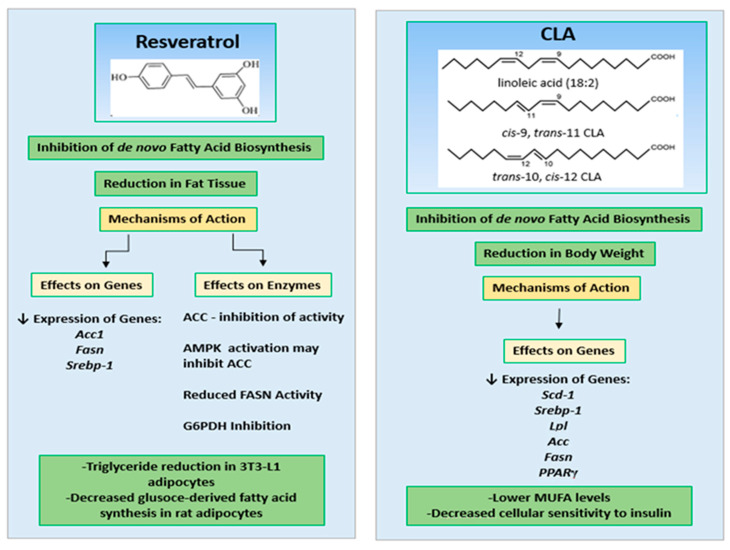
Structure of resveratrol, conjugated linoleic acid (CLA) and its isomers and possible mechanism of action on de novo fatty acids biosynthesis.

Resveratrol is a polyphenol with a stilbene structure, mainly found in grapes (*Vitis vinifera* L.) and in products made from them—in particular, wine [16]. Based on EU regulation resveratrol is recognized as the novel food [17]. Similar findings have been discovered for CLA, an organic chemical compound from the group of unsaturated fatty acids [18]. In addition, the EFSA assessed the safety of the CLA used in food products (EFSA Panel on Dietetic Products, Nutrition and Allergies (NDA)). Both resveratrol and CLA are considered safe natural substances with high functional/healthy properties [19]. Conjugated linoleic acid (CLA) is produced mainly by *Butyrovibrio fibrisolvens* and other anaerobic bacteria. These bacteria inhabit the rumen of cattle, as well as goats and sheep. The process in which CLA is produced involves the biohydrogenation of other polyunsaturated fatty acids [20,21], e.g., *trans-11* stearic acid [22]. Both bioactives are reported as natural substances for modulated lipid metabolism, including fatty acid biosynthesis de novo (Figure 1) [15,16,21,22].

The aim of this study was to investigate the effects of resveratrol, *cis-9*, *trans-11* CLA, *trans-10*, *cis-12* CLA, and, for the first time, their different combinations on the expression of selected genes associated with the de novo fatty acid biosynthesis process (fatty acid synthase-*Fasn*, acetyl-CoA carboxylase-*Acc1*, ATP citrate synthase-*Acly*, protein kinase AMP-activated catalytic subunit alpha 1 and 2-*Prkaa1* and *Prkaa2*, protein kinase CAMP-activated catalytic subunit alpha-*Prkaca*, sterol regulatory element-binding transcription factor 1-*Srebp1*) and how changes in mRNA levels correspond to the levels of specific proteins acetyl-CoA carboxylase 1 (ACC) and fatty acid synthase (FASN).

## 2. Results and Discussion

### Selection of the Optimal, Non-Toxic Concentration of the Tested Substances

**Figure 2:**
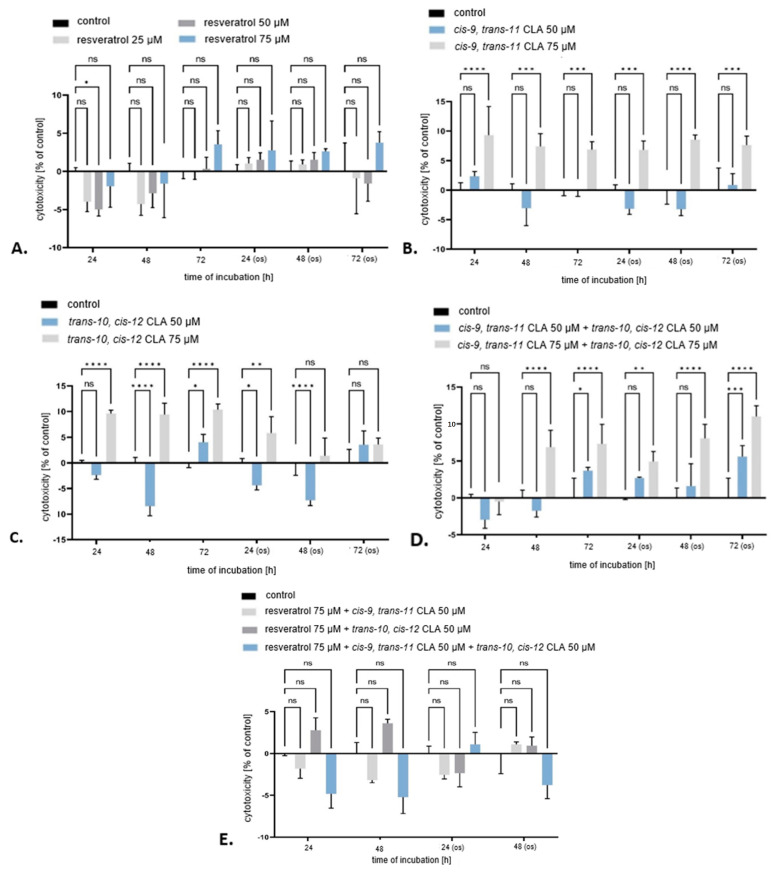
Determination of cytotoxicity in 3T3-L1 adipocytes due to the action of (**A**) 25, 50 and 75 µM resveratrol; (**B**) 50 and 75 µM *cis-9*, *trans-11* CLA; (**C**) 50 and 75 µM *trans-10*, *cis-12* CLA; (**D**) 50 and 75 µM mixture of *cis-9*, *trans-11* CLA, and *trans-10*, *cis-12* CLA; (**E**) mixtures of 75 µM resveratrol with 50 µM *cis-9*, *trans-11* CLA, 75 µM resveratrol with 50 µM *trans-10*, *cis-12* CLA, 75 µM resveratrol with 50 µM *cis-9*, *trans-11* CLA and 50 µM *trans-10*, *cis-12* CLA after 24, 48 and 72 h of incubation under standard conditions and AAPH-induced oxidative stress (os); *n* = 8. Statistical significance of differences measured by Student’s T-test (*p* < 0.05). (ns *p* > 0.05; * *p* < 0.05; ** *p* < 0.01; *** *p* < 0.001; **** *p* < 0.0001).

The cytotoxicity assessment of various combinations of substances on 3T3-L1 adipocytes provides clear insights into their safety. None of the tested substances exceeded a 20% increase in cytotoxicity within the range of concentrations and time points used. Resveratrol, in particular, showed no significant cytotoxic effects even at higher concentrations (Figure 2), confirming its well-documented safety and beneficial effects. Resveratrol is widely recognized for its antioxidant, anti-inflammatory, and anticancer properties [23,24]. Its lack of cytotoxicity at 75 µM supports its potential for use in various therapeutic and nutritional applications without adverse effects on adipocyte viability. Conversely, the CLA isomers demonstrated varying degrees of cytotoxicity. The *cis-9*, *trans-11* CLA at 75 µM significantly increased lactate dehydrogenase (LDH) levels across all time points, indicating cell membrane damage both under standard conditions and AAPH-induced oxidative stress (Figure 2). These findings are in agreement with recent studies that have documented the cytotoxic and pro-apoptotic effects of high concentrations of *cis-9*, *trans-11* CLA in different cell types [25,26]. Similarly, *trans-10*, *cis-12* CLA exhibited cytotoxic effect, particularly at a concentration of 75 µM under standard conditions and at 50 µM after 72 h of incubation (Figure 2). Previous studies have also reported the cytotoxic effects of *trans-10*, *cis-12* CLA, but on cancer cell lines [27]. By contrast, research by Whigham et al. [28] considered *trans-10*, *cis-12* CLA to be safe and beneficial, without reporting significant cytotoxic effects at comparable concentrations. These discrepancies underscore the variability in experimental outcomes, depending on different cell models, conditions, and assessment methods.

Based on these cytotoxic profiles, the concentrations selected for further testing were 75 µM for resveratrol, 50 µM for *cis-9*, *trans-11* CLA, and 50 µM for *trans-10*, *cis-12* CLA. The selection criteria aimed to balance the necessity of observing bioactive effects without inducing excessive cytotoxicity. These findings contribute to a deeper understanding of the differential impacts of resveratrol and CLA isomers on adipocyte viability, with significant implications for their application in nutritional and therapeutic contexts.

LDH concentration increased in 3T3-L1 adipocytes treated with a mixture of *cis-9*, *trans-11* CLA with *trans-10*, *cis-12* CLA, at concentration of 50 μM, after 72 h under standard conditions and oxidative stress. Notably, this increase was consistently observed in all experiments using a 75 µM mixture of *cis-9*, *trans-11* CLA with *trans-10*, *cis-12* CLA, with the exception of 24 h under standard conditions (Figure 2). In turn, Domagała et al. [29] reported that a mixture of fatty acids containing CLA isomers had no toxic effect on the cancer cells of line WM793. Igarashi and Miyazawa [30] showed that the cytotoxic effect of CLA isomers on five different cancer cell lines, DLD-1 (colon adenocarcinoma), HepG2 (hepatocellular carcinoma), A549 (lung adenocarcinoma), MCF-7 (breast adenocarcinoma), and MKN-7 (gastric adenocarcinoma), depended on both the concentration of the CLA isomer mixture and the cell type. The viability of HepG2 cells was progressively reduced under the influence of CLA isomers at concentrations above 5 µM, A549 cells above 10 µM, MCF-7 cells above 25 µM, and MKN-7 cells above 100 µM. Interestingly, cells from the DLD-1 line were not sensitive to CLA in the concentration range tested. On the other hand, Bocca et al. [31] did not show any cytotoxic effect of the CLA mixture on Caco-2 colon cancer cells in the tested concentration range (1–60 µM). From the above literature, it can be concluded that the toxicity of fatty acids depends on their type and concentration, as well as on the type of cells analyzed.

Mixtures of 75 µM resveratrol with 50 µM *cis-9*, *trans-11* CLA, 75 µM resveratrol with 50 µM *trans-10*, *cis-12* CLA and 75 µM resveratrol with 50 µM *cis-9*, *trans-11* CLA and *trans-10*, *cis-12* CLA did not result in an increase in LDH levels (Figure 2). It is notable that there is no existing publication that specifically addresses the effects of these particular combinations of resveratrol and CLA isomers on LDH levels. This gap in the literature serves to highlight the novelty of these current findings.

Our results indicate that the tested substances can be safely used in further studies of gene expression and selected proteins. It was determined that due to the cytotoxic effect of tested substances after 72 h incubation of cells, biological material used to analyze the expression of selected genes and proteins were isolated after 24 and 48 h.

**Figure 3:**
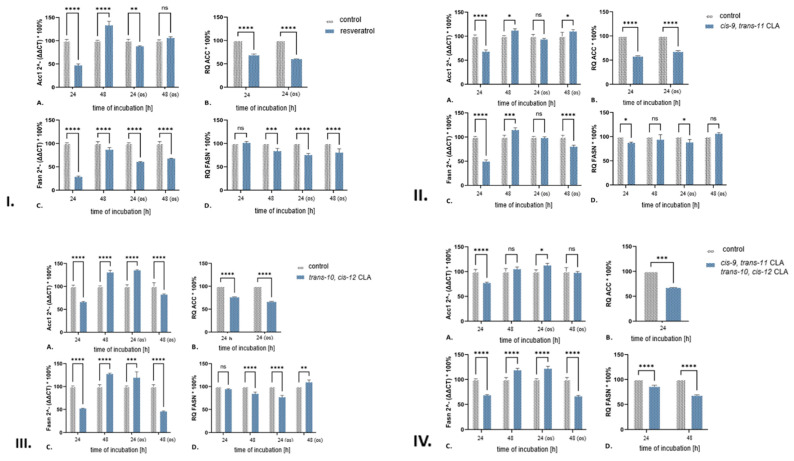
Expression of Acc1 and Fasn genes and ACC and FASN proteins in 3T3-L1 adipocytes treated with: (**I**). resveratrol (75 µM). (**II**). cis-9, trans-11 CLA (50 µM) (**III**). trans-10, cis-12 CLA (50 µM); (**IV**) a mixture of *cis-9*, *trans-11* CLA (50 µM) and *trans-10*, *cis-12* CLA (50 µM) under standard culture conditions and induced oxidative stress (os). Data are presented relative to controls. A. *Acc1* gene expression. B. Amount of ACC protein. C. *Fasn* gene expression D. FASN protein amount. *n* = 3. Statistical significance of differences measured by Student’s T-test (ns *p* > 0.05; * *p* < 0.05; ** *p* < 0.01; *** *p* < 0.001; **** *p* < 0.0001).

Resveratrol (trans-3,4′,5-trihydroxystilbene) treatment of 3T3-L1 adipocytes resulted in a significant decrease in the expression of genes related to de novo biosynthesis of fatty acids and selected proteins under both standard and oxidative stress (os) conditions. The effect was pronounced in cells with induced oxidative stress, as evidenced by the decrease in the content of ACC and FASN proteins (Figure 3). Expression of *Acc1*, *Fasn*, and *Srebp1* genes were differently in both experimental conditions (under standard and oxidative stress) at 24 and 48 h, as shown in Figure 3 and Table 1.

After 24 h of incubation with resveratrol, the number and size of lipid droplets in 3T3-L1 adipocytes were progressively reduced under standard conditions (Figure S1). These findings demonstrate a clear association between resveratrol and the reduction in lipid droplets in 3T3-L1 adipocytes. According to Peiva et al. [32], the reduction in lipid droplets can be explained by a decrease in the intensity of the de novo fatty acid biosynthesis process, which is consistent with a decrease in the amount of malonyl-CoA and acetyl-CoA, substrates required for the production of palmitate by FASN. The significant decrease in the expression of *Acc1* and *Acly* genes, as well as ACC protein after 24 h in standard conditions, obtained in our research, supports this conclusion (Figure 3, Table 1). Although there was notable down-regulation in *Fasn* expression (−71.4%) after 24 h, no significant changes in FASN protein content were observed (Figure 3). This lack of difference in the amount of fatty acid synthase is likely due to the long half-life of this protein in cells [33]. The accuracy of this assumption is supported by the results obtained after 48 h. *Fasn* gene expression decreased by 13.3%, and its protein content decreased by 16.3%. However, after 48 h, we observed an increase in expression of all tested genes (except the *Prkaa2* gene) relative to the discussed process (Figure 3, Table 1). This indicates that the effect of resveratrol on the tested cells disappears over time. The increase in the expression of the *Srebp1* gene in the examined cells after 48 h explained the increased transcription of *Acc1* and *Acly* (Figure 3, Table 1). The process of de novo fatty acid biosynthesis could be controlled by the *Srebp1* gene and also regulated by the protein kinases AMPK and PKA. A decrease in *Prkaca* expression suggests a potential weakening of ACLY protein activity due to reduced activation of this enzyme by PKA [34].

Shih et al. [35] demonstrated that treatment with resveratrol significantly reduced the expression of *Fasn* in 3T3-L1 cells differentiating into adipocytes. Fischer-Posovszky et al. [36] showed a significant decrease in the *Fasn* mRNA level in human adipocyte cells treated with 20–100 µM resveratrol. Our research showed a significant decrease in *Fasn* expression at the mRNA level, which is not consistent with the data obtained by Lasa et al. [37], who did not observe the effect of the resveratrol (at concentrations of 10 and 100 µM) on the expression of *Fasn*.

This study demonstrates that resveratrol has a significant impact on de novo fatty acid biosynthesis in 3T3-L1 adipocytes under oxidative stress conditions. The results indicated a decrease in the expression of the *Acc1* gene (−11.9%) and the ACC protein (−40%) after 24 h of incubation. This reduction in the cells’ ability to produce malonyl-CoA was also observed under standard conditions. In contrast to the decrease in *Acly* gene expression observed under standard conditions, induced oxidative stress resulted in an increase in the expression of this gene in 3T3-L1 adipocytes (Table 1). This suggested an increase in the availability of the second substrate required for FASN, namely acetyl-CoA. Resveratrol retained its properties related to the de novo fatty acid biosynthesis process, even under oxidative stress conditions. This could result in the deficiency of one of the substrates necessary for FASN and a significant decrease in the amount of this enzyme (FASN; −24.8%) and its mRNA (Figure 3).

The recorded level of *Acc1* mRNA indicated that there was no weakening of malonyl-CoA synthesis after incubating the tested cells with resveratrol for 48 h under oxidative stress conditions (Figure 3). It is worth noting that a decrease in *Acly* expression is likely associated with a decrease in the availability of acetyl-CoA, derived from citrate breakdown, in 3T3-L1 adipocytes. The reduction in malonyl-CoA synthesis could be compensated for by the synthesis of acetyl-CoA from acetate, catalyzed by ACSS1, or by peroxisomal β-oxidation [38]. It is likely that after 48 h, there was further inhibition of the de novo fatty acid biosynthesis process, even though the reduction in malonyl-CoA synthesis was not demonstrated after 24 h. There was a reduction in the amount of FASN protein by 19.4% and in the corresponding mRNA level by 32.4%. 

There was no significant difference in *Srebp1* gene expression compared to the control after a 48 h of incubation (condition of oxidative stress). The obtained results for the expression of *Prkaa1* and *Prkaa2* genes indicate that the reduced expression of these genes can be attributed to the inhibition of SREBP1 activity by AMPK. AMPK and PKA kinases have a similar effect on ACC protein. The increase in *Prkaca* expression indicates increased phosphorylation of ACC by both AMPK and PKA, resulting in a decrease in the intensity of malonyl-CoA production [39]. 

Resveratrol found in natural sources such as red grapes, berries, peanuts, and dark chocolate can regulate key enzymes and signaling pathways involved in lipid metabolism, helping to reduce fat accumulation and support metabolic health [40]. Despite low intestinal absorption, which causes the low levels of resveratrol found in plasma and tissues, the beneficial effects of this compound have been repeatedly described in the literature [41]. Although the mechanism of action of resveratrol is still not fully understood, probably due to the pleiotropic effect of this compound, many authors have shown that this compound can cause a reduction in adipocyte proliferation, a decrease in lipogenesis, and also increased apoptosis and lipolysis in adipose tissue, in addition to indirectly enhancing fatty acid oxidation in liver and skeletal muscle through mitochondriogenesis [41,42,43]. Da Costa et al. [44] showed that feeding mice a high-fat diet with grape skin extract enriched in polyphenolic fractions reduced epididymal and retroperitoneal fat mass, lipid profile, glucose, and insulin levels as compared to animals fed a high-fat diet. In addition, Bedê et al. [45] reported that rats fed a high-fat diet with grape juice had significantly lower levels of interleukin-6, total cholesterol, and triglycerides than rodents fed the same diet supplemented with red wine or resveratrol.

Multiple studies, including those using cell models or animals, have demonstrated the significant impact of CLA on fat tissue deposition and lipid content in the body [46,47,48]. CLA may have potential as a nutrient for humans, but further research is needed to fully understand its effectiveness. Studies on CLA using a cellular model focus on preadipocytes during differentiation, while only a few investigate the effect of *cis-9*, *trans-11* CLA on gene expression related to fat metabolism. Brown et al. [49,50] found that *cis-9*, *trans-11* CLA, as opposed to *trans-10*, *cis-12* CLA, increases triacylglyceride accumulation and adipocyte-specific gene expression in human fat cells. 

Our research indicated a decrease the number and size of lipid droplets in 3T3-L1 adipocyte treatment with *cis-9*, *trans-11* CLA (Figure S2). Under standard conditions, treatment with the mentioned isomer of CLA significantly inhibited the process of de novo fatty acid biosynthesis in 3T3-L1 adipocytes. After 24 h of incubation, the decrease in FASN protein amount (12.9%) was likely due to a deficiency of malonyl-CoA and acetyl-CoA, as indicated by the decrease in ACC protein amount (−42.6%) and *Acc1* and *Acly* mRNA (Figure 3, Table 1). Table 1 shows an association between decreased *Srebp1* mRNA levels and the expression of its target genes *Acc1*, *Fasn*, and *Acly*. It is worth noting that the reduction in the phosphorylation of proteins formed on the matrix of the aforementioned genes was associated with a decrease in the level of the AMPK and PKA proteins. However, these relationships were not observed after 48 h of treatment with 3T3-L1 *cis-9*, *trans-11* CLA (Table 1). The studies conducted by Cordoba-Chacon et al. [48] and Tsuboyama-Kasaoka et al. [46] showed a significant decrease in the expression of *Fasn* and *Acc1* in the adipose tissue of C57BL/6J mice after being fed with the diet supplemented with *cis-9*, *trans-11* CLA. These findings provide evidence for the effectiveness of the diet in reducing the expression of these genes. 

No studies have been conducted on the effect of *cis-9*, *trans-11* CLA isomer on the expression of genes and the content of proteins related to de novo fatty acid biosynthesis in 3T3-L1 adipocytes under conditions of oxidative stress. Isomer *cis-9*, *trans-11* CLA has been shown to attenuate oxidative stress induced by LPS in bovine mammary epithelial cells, resulting in increased fat production [51]. 

This study reported a significant decrease in the expression of ACC and FASN proteins in 3T3-L1 adipocytes in both experimental systems. Moreover, a notable reduction in mRNA levels for *Acc1*, *Fasn*, *Acly*, *Srebp1*, *Prkaa2*, and *Prkaca* genes was observed under standard conditions. It is worth noting that in cells with induced oxidative stress, only the expression of the *Srebp1* gene was reduced (Table 1).

After incubating 3T3-L1 adipocytes with *cis-9*, *trans-11* CLA for 24 h under oxidative stress conditions, a decrease in the amount of ACC protein was observed (Figure 3). However, no significant changes in mRNA levels for *Acc1* and *Fasn* were detected. The difference can be attributed to the earlier suppression of gene expression or potential regulation at the protein translation level. This is linked to the reduction in mTORc1 or eIF6 activity, as suggested by Düvel et al. [52] and Brina et al. [53].

After 48 h of incubation of 3T3-L1 adipocytes with *cis-9*, *trans-11* CLA, an increase in *Acc1* and *Fasn* expression was observed. Additionally, induced oxidative stress led to reduced levels of *Fasn* and *Srebp1* mRNA in the cells. It is worth noting that the quantity of FASN protein did not differ from the control in both experimental systems (Table 1). The data demonstrated a significant reduction in the concentration of *cis-9*, *trans-11* CLA in cells under standard conditions due to its metabolic breakdown. Furthermore, induced oxidative stress in 3T3-L1 adipocytes resulted in a notable inhibition and delay of *cis-9*, *trans-11* CLA metabolism. 

Ma et al. [51] showed a significant increase in the expression of *Fasn*, *Acc1*, and *Srebp1* genes in bovine mammary epithelial cells (BMECs) after 24 h of incubation with 50 µM *cis-9*, *trans-11* CLA under oxidative stress conditions. However, after 48 h of incubation with this CLA isomer (50 and 100 µM), there was a significant decrease in the expression of these genes. These results suggest that the effects of *cis-9*, *trans-11* CLA on gene expression may be time and dose dependent.

Treatment with *trans-10*, *cis-12* CLA for 24 h resulted in a decrease in de novo fatty acid biosynthesis intensity in 3T3-L1 adipocytes without induced oxidative stress, similar to the experiment with *cis-9*, *trans-11* CLA (Figure S3). The expression of the examined genes (*Acc1* and *Acly*) showed analogous changes in both cases, leading to a decrease in the substrates required for FASN to synthesize palmitate. The application of *trans-10*, *cis-12* CLA resulted in a reduction in ACC content in the examined cells (Figure 3). Although there were no significant differences in FASN quantity (Figure 3). It can be suggested that the decrease in the intensity of the process was solely due to reduced synthesis of acetyl-CoA and malonyl-CoA, unlike *cis-9*, *trans-11* CLA. 

The examination of genes related to the discussed process revealed an intensification in their expression after 48 h due to the action of *trans-10*, *cis-12 CLA* (Figure 3 and Table 1). It is noteworthy that this effect was observed in almost all genes marked within the conducted research, unlike the experiments using *cis-9*, *trans-11* CLA. The observed decrease in the FASN protein level (−15.8%) in the examined genes could result in a reduction in de novo fatty acid biosynthesis. 

The analysis of gene expression results related to the regulation of the discussed process showed that changes in the expression of *Acc1*, *Fasn*, and *Acly* corresponded to changes marked for *Srebp1* at both time points (Table 1). As demonstrated by Göransson et al. [54], adipocytes treated with *trans-10*, *cis-12* CLA showed activation of AMPK. The study of gene expression suggests a possible decrease in the quantity of AMPK. However, it is important to note that this did not rule out an increase in its activity. Furthermore, a decrease in the expression of *Prkaca* was observed after 24 h, along with a decrease in phosphorylation potential towards ACC and SREBP1. Koeberle and Su [55], Li et al. [56], and Lu and Shy [57] have provided detailed information on the interactions between AMPK and PKA, and ACC and SREBP1, as described above. 

The results obtained in 3T3-L1 preadipocytes treated with 50 µM *trans-10*, *cis-12* CLA were found to be consistent with those previously reported by den Hartigh et al. [58]. Our research demonstrated a significant decrease in *Acc1* expression and no effect on *Fasn* transcription. Brown et al. [50] showed a significant reduction in *Acc1* expression in human preadipocytes treated with the discussed CLA isomer (30 µM). This finding is further supported by Cordoba-Chacon et al. [48], who identified a decrease in *Acc1* and *Fasn* expression after 24 h. The study demonstrated a significant reduction in the expression of the genes in the inguinal white and brown adipose tissue of C57BL/6J mice after being fed a diet supplemented with 0.8% *trans-10*, *cis-12* CLA.

The results indicated that induced oxidative stress led to a significant decrease in de novo fatty acid biosynthesis in cells treated with *trans-10*, *cis-12* CLA for 24 h. This decrease was more pronounced compared to the results obtained under standard culture conditions, as evidenced by the reduction in ACC and FASN protein levels. It is worth noting that the reduced levels of these proteins in the examined cells did not align with the expression of genes associated with them, which increased despite the decrease in *Srebp1* (Figure 3 and Table 1). This disparity between gene expression levels and the quantity of corresponding proteins is likely due to regulation at the level of translation. 

The impact of *trans-10*, *cis-12* CLA on FASN levels may be complex, depending on the cellular environment. However, an increase in FASN levels was observed in cells with induced oxidative stress, indicating intensification of de novo fatty acid biosynthesis. By contrast, cells cultured under standard conditions demonstrated a further decrease in the quantity of this protein, suggesting a persistent weakening of de novo fatty acid biosynthesis. The gene expression values obtained under induced oxidative stress indicated the opposite situation over a time period exceeding 48 h (Figure 3 and Table 1).

The inhibitory effect of a mixture of *cis-9*, *trans-11* and *trans-10*, *cis-12* CLA on the activity of stearoyl-CoA desaturase in the white adipose tissue of BALB/c mice fed with a diet supplemented with 1% of the isomers has been demonstrated by Jaudszus et al. [59]. Arias et al. [60] showed that the addition of a mixture of *cis-9*, *trans-11* CLA isomers and *trans-10*, *cis-12* CLA to a high-fat and high-calorie diet led to a significant reduction in adipose tissue in Wistar rats, without any accompanying changes in body weight.

The combination of *cis-9*, trans-11 CLA and *trans-10*, *cis-12* CLA isomers decreased the intensity of de novo fatty acid biosynthesis (Figure S4). The reduction was due to a decrease in the amount of FASN and malonyl-CoA proteins in the tested cells, as well as a decrease in *Srebp1* gene expression. These findings are consistent with the results of Tsuboyama-Kasaoka et al. [30,46], who demonstrated a decrease in the expression of *Acc1*, *Fasn*, and *Srebp1* in the adipose tissue of C57BL/6J mice fed with a diet supplemented with 1% of a mixture of CLA isomers. The study presented findings indicating that the mixture of CLA isomers had a moderate impact on ACC, which was stronger than that of *trans-10*, *cis-12* CLA but weaker than that of *cis-9*, *trans-11* CLA (Figure 3). A mixture of the studied CLA isomers resulted in an intensification of acetyl-CoA synthesis from citrate after 24 h, as evidenced by an increase in *Acly* expression (Table 1 ). These findings suggest that the studied CLA isomers have a differential impact on gene expression. Arias et al. [60] obtained an antagonistic effect of the isomers of CLA used in the mixture regarding the reduction in adipose tissue size in rats.

Treatment with a mixture of CLA isomers for 48 h resulted in increased expression of *Acc1*, *Fasn*, *Acly*, *Srebp1*, and *Prkaca* genes in adipocytes (Table 1). The decrease in the concentration of the tested mixture due to its metabolism may have contributed to this increase. This observation is consistent with the findings of Shen et al. [61]. They reported a reversed dose-dependency effect, where lower concentrations of the CLA isomer mixture resulted in a stronger increase in gene expression than higher concentrations.

After 24 h of cell treatment with a mixture of CLA isomers under oxidative stress conditions, the expression of *Fasn* and *Acly* genes increased and then decreased after 48 h, while the mRNA content of *Srebp1* decreased progressively over time. These results differ from those obtained under standard culture conditions. The mixture of CLA isomers was capable of decreasing the intensity of the fatty acid biosynthesis process in 3T3-L1 adipocytes under oxidative stress conditions. However, it is important to note that the decreases in gene expression observed for the mixture of CLA isomers were lower than when using only *trans-10*, *cis-12* CLA and higher than in experiments where cells were treated with *cis-9*, *trans-11* CLA, which suggests an antagonistic effect.

**Figure 4:**
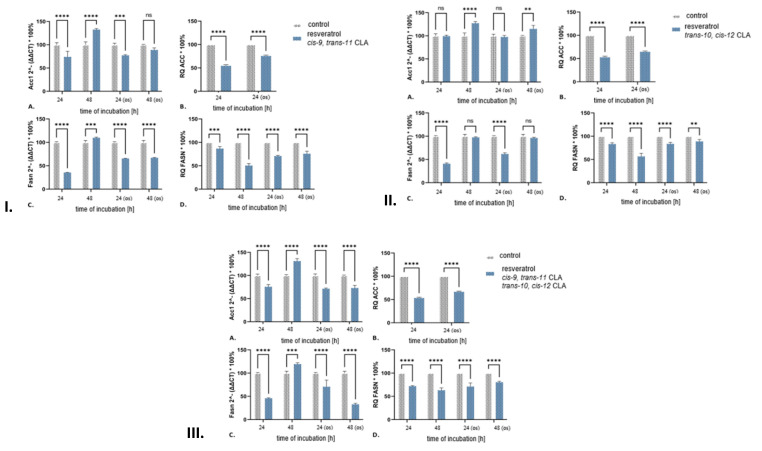
Expression of Acc1 and Fasn genes and ACC and FASN proteins in 3T3-L1 adipocytes treated with (**I**) a mixture of resveratrol (75 µM) and *cis-9*, *trans-11* CLA (50 µM); (**II**) a mixture of resveratrol (75 µM) and *trans-10*, *cis-12* CLA (50 µM); (**III**) an equimolar mixture of resveratrol, *cis-9*, trans-11 CLA, and trans-10, cis-12 CLA under standard culture conditions and induced oxidative stress (os). Data are presented relative to controls. A. Acc1 gene expression. B. Amount of ACC protein. C. *Fasn* gene expression D. FASN protein amount. *n* = 3 Statistical significance of differences measured by Student’s T-test (ns *p* > 0.05; ** *p* < 0.01; *** *p* < 0.001; **** *p* < 0.0001).

This study found that treatment with a mixture of *cis-9*, *trans-11* CLA and *trans-10*, *cis-12* CLA under oxidative stress conditions for 24 h led to an increase in the expression of *Prkaa1*, *Prkaa2* and *Prkaca* genes in 3T3-L1 adipocytes. This increase suggested a rise in the phosphorylation potential of AMPK and PKA, which may have contributed to the decrease in ACC and SREBP activity. The gene expression values for AMPK obtained from the experiment using the CLA isomer mixture were consistent with those obtained when the tested cells were exclusively treated with *trans-10*, *cis-12* CLA. A decrease in the expression of *Prkaa1*, *Prkaa2*, and *Prkaca* genes at the second time point led to an increase in ACC and SREBP activity. The reduction in *Prkaca* expression in cells treated with a combination of the tested CLA isomers was lower than that observed when only *trans-10*, *cis-12* CLA was used but higher than the reduction observed in the experiment using *cis-9*, *trans-11* CLA (Table 2 and Figure 4).

Studies have not extensively explored the effects of combining resveratrol and *cis-9*, *trans-11* CLA. However, Arias et al. [60] demonstrated the efficacy of CLA in reducing adipose tissue in adult Wistar rats that were fed with diet TD.06415 and supplemented with 0.5% CLA for 6 weeks. 

Microscopic observation indicated a progressive reduction in the number and size of lipid droplets in the examined cells after treatment with resveratrol and *cis-9*, *trans-11* CLA (Figure S5). The tested cells incubated with the mentioned mixture showed a decrease in ACC levels (−45%), which was comparable to the effect demonstrated by the action of *cis-9*, *trans-10* CLA and higher than in the case of resveratrol alone (Figure 3 and Figure 4). The reduction in palmitate synthesis observed in cells treated with a combination of resveratrol and *cis-9*, *trans-11* CLA can be attributed to the decreased availability of malonyl-CoA. This, in turn, resulted in a reduction in palmitate synthesis due to the decreased level of FASN. This effect was not observed in cells treated solely with resveratrol (Figure 3). The cause of the described changes was determined to be related to the action of SREBP1 proteins, as evidenced by the decrease in mRNA levels in the tested cells (Table 2). This decrease was less pronounced in 3T3-L1 adipocytes treated with a mixture of resveratrol and *cis-9*, *trans-11* CLA, compared to experiments using individual components (Table 1 and Table 2). After 24 h, there was a significant increase in the expression of *Prkaa1* and *Prkaa2* genes, accompanied by a notable decrease in *Prkaca* (Table 2). These findings suggested that AMPK plays a crucial role in controlling the activity of ACC and SREBP, leading to a significant weakening of de novo fatty acid biosynthesis processes in the cells [62,63,64]. This is a difference compared to the results obtained in experiments using individual components of the discussed mixture (Table 1), where either no differences in *Prkaa1* and *Prkaa2* gene expression were observed (resveratrol) or a decrease in the expression of one of them was demonstrated (*cis-9*, *trans-11* CLA). All experimental conditions consistently demonstrated a decrease in *Prkaca* expression, providing evidence for a weakening of the inhibitory phosphorylation activity of ACC by PKA [58]. However, it is possible that the action of AMPK on ACC is counteracted by PKA. Inhibiting the activity of AMPK is more likely to result in a decrease in *Fasn* expression due to its indirect interaction through SREBP1 [63].

The combination of resveratrol and *cis-9*, *trans-11* CLA has a significant impact on 3T3-L1 adipocytes under standard conditions after 48 h. Microscopic observation clearly showed a gradual reduction in both the number and size of lipid droplets in the tested cells (Figure S5). This effect was due to the significant inhibition of palmitate synthesis by FASN (−49.5%) (Figure 4). Moreover, despite the increase in *Fasn* mRNA levels and the expression of *Acc1*, *Srebp1*, and *Acly* genes (Table 2), a decrease in FASN protein level was observed. This decrease could be attributed to the significant decrease in the *Fasn* mRNA level after 24 h, which was also observed in experiments where cells were treated solely with resveratrol or *cis-9*, *trans-11* CLA (Table 1). It seems like AMPK inhibits SREBP1 activity through the upregulation of *Prkaa1* and *Prkaa2* [65]. Treatment of adipocytes with resveratrol alone and resveratrol with *cis-9*, *trans-11* CLA resulted in an increase in *Prkaa1* and *Prkaca* expression after 48 h (Table 1 and Table 2), intensifying the phosphorylation potential of AMPK and PKA. This may lead to the inhibition of ACC activity and reduce the availability of malonyl-CoA in cells [66]. 

Treatment of 3T3-L1 adipocytes with a combination of resveratrol and *cis-9*, *trans-11* CLA for 48 h resulted in a significant increase in *Acly* and *Prkaca* transcription, comparable to the effects of resveratrol and *cis-9*, *trans-11* CLA individually (Table 1 and Table 2). Both resveratrol and *cis-9*, *trans-11* CLA enhanced *Acc1* expression after 48 h. This indicated a potential synergistic effect when used in combination, although further confirmation is required. Notably, the increase in *Acc1* expression was equivalent to that observed in the experiment with resveratrol alone. The increase in mRNA content of *Srebp1* was significantly higher after 48 h of incubation with the mixture than what could be expected from data obtained for resveratrol and *cis-9*, *trans-11* CLA alone. This suggests that the effects of resveratrol and *cis-9*, *trans-11* CLA on *Fasn* expression are counteracted by each other in the mixture (Table 1 and Table 2). 

The application of a mixture of resveratrol and *cis-9*, *trans-11* CLA resulted in a reduction in de novo fatty acid biosynthesis under oxidative stress conditions. Exposure of cells to induced oxidative stress resulted in a reduction in the expression level of genes and tested proteins (Table 2). The observed increase in *Prkaa1* and *Prkaa2* gene expression after 24 h of treatment with a mixture of resveratrol and *cis-9*, *trans-11* CLA in 3T3-L1 adipocytes under standard conditions suggested that AMPK activity inhibited ACC and SREBP activity.

Adipocytes treated with a combination of resveratrol and *cis-9*, *trans-11* CLA exhibited decreased expression of *Acc1* and *Fasn* under oxidative stress conditions, with no significant differences in the expression of their controller, *Srebp1* (Figure 4, Table 2). This study demonstrated a significant reduction in the levels of ACC and FASN proteins. These results provide evidence for the weakening of the intensity of de novo fatty acid biosynthesis processes in 3T3-L1 adipocytes. This is likely due to both reduced malonyl-CoA synthesis and a decrease in fatty acid synthase content.

Under conditions of oxidative stress, both resveratrol and *cis-9*, *trans-11* CLA caused a significant decrease in the levels of ACC and FASN proteins in 3T3-L1 adipocytes after 24 h. This decrease was not aligned with changes in *Acc1* and *Fasn* gene expression (Table 1 and Table 2). The combination of resveratrol and *cis-9*, *trans-11* CLA resulted in a lower decrease in FASN protein level and *Acc1* mRNA in 3T3-L1 adipocytes after 48 h compared to the individual components. The decrease in *Fasn* expression in cells treated with the mixture was similar to the values obtained in the experiment with resveratrol alone. The application of a combination of resveratrol and *cis-9*, *trans-11* CLA during oxidative stress conditions is likely to result in a reduction in the intensity of the de novo fatty acid biosynthesis process, which is consistent with the effect of resveratrol alone. However, the *cis-9*, *trans-11* CLA isomer in the mixture did not significantly impact the changes in *Acc1* and *Fasn* expression. These findings suggested that resveratrol was the key component responsible for the observed effects. Differences in the expression of *Prkaa1* and *Prkaa2* indicated a more effective inhibition of ACC and SREBP1 activity compared to using resveratrol alone (Table 1 and Table 2).Kennedy et al. [66] established a relationship between resveratrol and *trans-10*, *cis-12* CLA in human adipocytes. The study found that these substances attenuated each other’s effects. Specifically, resveratrol was found to increase intracellular Ca^2+^ concentrations, which in turn blocked the reduction in accumulated fat in adipocytes. Furthermore, resveratrol was observed to weaken the inhibition of Pparγ activity by *trans-10*, *cis-12* CLA. According to Sharma et al. [67], resveratrol enhanced insulin action. In a study conducted by Arias et al. [40] on adult Wistar rats fed a diet supplemented with resveratrol, *trans-10*, *cis-12* CLA (0.5%), or a mixture of the two for 6 weeks, it was found that resveratrol did not mitigate *trans-10*, *cis-12* CLA-induced insulin resistance.

Our results demonstrated that the mixture of resveratrol and *trans-10*, *cis-12* CLA inhibited the de novo fatty acid biosynthesis process in 3T3-L1 adipocytes. This inhibition was attributed to the reduced ability of 3T3-L1 adipocytes to synthesize malonyl-CoA, which was associated with a decrease in ACC levels (Figure 4). The increase in the synthesis of the second substrate for FASN is attributed to the increase in *Acly* expression. Notably, this increase was not observed as a result of the action of any of the individual components of the investigated mixture separately (Table 1 and Table 2). Furthermore, a decrease in FASN level was observed in cells treated with a mixture of resveratrol and *trans-10*, *cis-12* CLA (Figure 3 and Figure 4). After 48 h, the intensity of the de novo fatty acid biosynthesis process decreased (Figure S6). In the experiment with a mixture of resveratrol and *cis-9*, *trans-11* CLA (Figure 4), the tested cells showed a decrease in FASN level (−43.3%) (Figure 3 and Figure 4). This finding was consistent with the study by Lasa et al. [37], where no changes in *Fasn* expression were observed in 3T3-L1 adipocytes treated with resveratrol, *trans-10*, *cis-12* CLA or a mixture of these compounds.

The transcription of *Acc1*, *Fasn*, and *Acly* genes in 3T3-L1 adipocytes treated with resveratrol or *trans-10*, *cis-12* CLA corresponded to changes in the mRNA level of *Srebp1* under standard experimental conditions. This relationship was observed only in relation to *Fasn* expression after 24 h in the experiment with a mixture of resveratrol and *trans-10*, *cis-12* CLA (Figure 3 and 4, Table 1 and Table 2).

The inhibition of de novo fatty acid biosynthesis in the examined cells under standard conditions for 24 h was linked to the limitation of malonyl-CoA synthesis by ACC and a reduction in FASN protein level. The process was weakened due to the increased action of AMPK, resulting in decreased ACC and SREBP1 activity. After incubating 3T3-L1 adipocytes with a mixture of resveratrol and *trans-10*, *cis-12* CLA for a longer period, an increase in the expression of *Prkaa1* and *Prkaca* was observed (Table 2). This increase was lower than that detected in the experiment using only resveratrol but higher than that induced by *trans-10*, *cis-12* CLA (Table 1 and Table 2). These findings suggest a potential attenuation of SREBP1 and ACC activity due to the actions of both AMPK and PKA.

The comparison of the expression levels of *Acc1* and *Fasn* genes obtained from experiments using resveratrol or *trans-10*, *cis-12* CLA separately and a mixture of these suggested antagonism between these two compounds (Table 1 and Table 2). However, the measured level of proteins resulting from the transcription of these genes indicated significant synergy (Figure 3 and Figure 4). Moreover, the experiment showed an intensification of *Prkaa1* and *Prkaa2* expression after 24 h in the case of the mixture, which was not present or even reduced in 3T3-L1 adipocytes treated solely with resveratrol or *trans-10*, *cis-12* CLA (Table 1 and Table 2). Additionally, there were no significant differences observed in the decrease in ACC and FASN proteins levels when compared to the results obtained in the experiment using a mixture of resveratrol with *cis-9*, *trans-11* CLA (Figure 4). The increase in *Prkaa1* and *Prkaa2* expression observed in cells treated with a combination of resveratrol and *trans-10*, *cis-12* CLA after 24 h was significantly higher than that observed with the combination of the studied resveratrol and *cis-9*, *trans-11* CLA (Table 2). Therefore, the use of resveratrol mixtures with each of the CLA isomers tested in this work results in a reduction in the levels of ACC and FASN proteins.

The application of a mixture of resveratrol and *trans-10*, *cis-12* CLA during oxidative stress weakened the intensity of the de novo fatty acid biosynthesis process. This is supported by changes in the expression of *Acc1*, *Fasn*, and *Srebp1* genes, as well as the levels of ACC and FASN proteins (Figure 4, Table 2). The experiment demonstrated that induced oxidative stress led to lower *Srebp1* gene expression and ACC protein levels after 24 h, as well as an increase in Acc1 expression and a decrease in FASN levels after 48 h in 3T3-L1 adipocytes (Figure 4, Table 2). The results showed a difference from the standard conditions, where the combination of resveratrol and trans-10, cis-12 CLA exhibited a more pronounced inhibitory effect on the de novo fatty acid biosynthesis process in 3T3-L1 adipocytes. 

The expression of the *Acc1* gene did not show any significant changes in both experimental conditions after 24 h compared to the control. However, the protein level produced from its matrix (ACC) was significantly lower compared to the control (−47.2% and −35.4%). The difference between mRNA levels and protein quantity may indicate strong inhibition of this gene’s transcription in the first 24 h of incubation. According to Damiano et al. [68], the protein has a half-life of approximately 6 h in cells, suggesting that the expression levels of *Acc1* will return to levels comparable to those of the control cells at the time of genetic material isolation. The observed discrepancy could also be explained by the control of ACC translation, resulting in a decrease in its synthesis in the examined cells.

The results obtained from experiments using a mixture of resveratrol and *trans-10*, *cis-12* CLA under induced oxidative stress were consistent with those obtained from experiments using only resveratrol or *trans-10*, *cis-12* CLA. Notably, no changes in the expression of *Acc1* and *Fasn* were observed in cells treated with the mentioned mixture, indicating that the mixture did not have any additional effect on the expression of these genes. Furthermore, after 24 h, a similar decrease in *Fasn* expression was observed in cells treated with the mentioned mixture and resveratrol alone. A significant decrease in transcription was observed in cells treated solely with resveratrol and *trans-10*, *cis-12* CLA (Table 1 and Table 2). However, the mRNA content of this gene did not differ significantly from the control after 48 h. In 3T3-L1 adipocytes, a comparable decrease in ACC quantity was observed in cells treated solely with resveratrol or *trans-10*, *cis-12* CLA, as well as their mixture, while the level of FASN remained unchanged. The results of the experiments with 3T3-L1 adipocytes incubated with a mixture of resveratrol and *trans-10*, *cis-12* CLA for 24 h, demonstrated a decrease in the level of FASN compared to experiments with individual components (Figure 3 and Figure 4).

In summary, the presence of *trans-10*, *cis-12* CLA in the mixture weakened the effect of resveratrol alone on the expression of *Acc1* and *Fasn*, as well as on the content of the FASN protein. The overall impact of the mixture of resveratrol with *trans-10*, *cis-12* CLA appeared to be weaker compared to the results obtained under standard culture conditions (Table 2). The combination of resveratrol with *cis-9*, *trans-11* CLA was found to be more effective in inhibiting the de novo fatty acid biosynthesis process in 3T3-L1 adipocytes under oxidative stress conditions than the combination of resveratrol with *trans-10*, *cis-12* CLA (Table 2).

Treatment with a combination of resveratrol, *cis-9*, *trans-11* CLA, and *trans-10*, *cis-12* CLA effectively reduced the intensity of de novo fatty acid biosynthesis in 3T3-L1 adipocytes under standard conditions (Figure S7). This was demonstrated by the observed decrease in malonyl-CoA and palmitate synthesis in the examined cells, which is consistent with previous experiments. The decrease in ACC and FASN proteins levels, as demonstrated in Figure 4, was a direct result of this mixture. It is important to highlight that FASN was affected by the mixture. Arias et al. [60] demonstrated that the activity of an enzyme in the perirenal adipose tissue of Wistar rats decreased when fed a diet containing a mixture of resveratrol (30 mg/kg body weight/day) and CLA (0.5%, equimolar mixture of *cis-9*, *trans-11* and *trans-10*, *cis-12*) for 6 weeks. However, it is important to note that the decrease was less significant than when the animals were fed a diet supplemented with resveratrol alone. The decrease in FASN levels in this study demonstrates that the discussed mixture weakens the synthesis of new fatty acids, as confirmed by Arias et al. [60]. Furthermore, 3T3-L1 adipocytes exhibited a weakening of this process after 48 h.

Treatment with a combination of resveratrol, *cis-9*, *trans-11* CLA, and *trans-10*, *cis-12* CLA for 24 h under standard conditions significantly decreased mRNA levels for *Srebp1* in 3T3-L1 adipocytes. This decrease led to a subsequent reduction in the transcription of *Acc1* and *Fasn*, as demonstrated in Figure 4. The outcomes of this study were in alignment with those of previous investigations into the substances and their mixtures. An increase in the expression of *Acly* was observed, similar to the experiment where cells were treated with a combination of resveratrol and *trans-10*, *cis-12* CLA. It is important to note that after 48 h, the expression of all genes crucial for fatty acid biosynthesis, including *Acc1*, *Fasn*, *Srebp1*, and *Acly*, significantly increased (Figure 4, Table 2). The observed increase in *Fasn* expression did not correspond to the amount of the protein it encodes. However, this discrepancy can be explained by a significant decrease in its expression due to the action of the tested mixture of bioactive substances after 24 h or the control of transcription factor translation, such as SREBP1 by eIF6 or the mTORC1 complex controlling transcription and translation processes in cells [52]. Brina et al. [53] declared that eIF6 enhances the activity of enzymes involved in fatty acid and cholesterol synthesis, while its absence leads to reduced cellular protein synthesis rates. Additionally, Ben-Sahra and Manning [69] stated that mTORC1 governs protein synthesis, ATP levels, and redox balance by integrating various cellular signaling pathways.

Our experiment demonstrated a significant increase in the expression of *Prkaa1* and *Prkaa2*, resulting in a marked intensification of AMPK activity and a subsequent decrease in ACC and SREBP activity. The observed increase in gene expression related to both AMPK and PKA after 48 h provided evidence for the regulation of the discussed process through ACC and SREBP phosphorylation. The results of gene expression related to AMPK for all tested substances and their combinations demonstrated synergy after 24 h under standard conditions in all mixtures containing resveratrol and CLA. Resveratrol’s ability to activate AMPK by inhibiting ATP production in mitochondria has been extensively studied and well documented in the literature [70,71]. CLA activates AMPK, although the mechanism of action is unclear, similar to resveratrol [72,73]. The study showed that the tested mixtures of resveratrol and CLA increase *Prkaa1* and *Prkaa2* gene expression, leading to the inhibition of de novo fatty acid biosynthesis processes.

The cells treated with the discussed mixture and each of its components separately exhibited a synergistic effect (at 24 h), as well as an additive effect (at 48 h), on the amount of FASN protein. Additionally, each of the three combinations of the substances used showed a similar impact on malonyl-CoA synthesis. This study confirmed that the mixture of resveratrol, *cis-9*, *trans-11* CLA, and *trans-10*, *cis-12* CLA is significantly more effective in inhibiting the de novo fatty acid biosynthesis process than its individual components. The comparison of the effects exerted by the discussed mixture and its individual components on *Acc1* expression indicated an antagonistic effect at 24 h. Notably, the decrease obtained for the mixture is lower than for each substance individually.

Inducing cells with oxidative stress and treating them with a mixture of resveratrol, *cis-9*, *trans-11* CLA, and *trans-10*, *cis-12* CLA resulted in changes related to the de novo fatty acid biosynthesis process. The observed changes include a decrease in ACC protein quantity and a reduction in the expression of *Acc1*, *Acly*, *Fasn*, and *Srebp1* genes (Figure 4 and Table 2). The results obtained led to the conclusion that the mixture’s action under oxidative stress conditions rapidly and durably inhibits the de novo fatty acid biosynthesis process.

Table 2 demonstrates a significant increase in *Prkaa2* expression and a decrease in ACC and FASN mRNA and protein content after 24 h under oxidative stress conditions. This indicated a stronger regulation of ACC and SREBP through AMPK phosphorylation. Additionally, it was possible that gene expression was controlled through a decrease in mTORC2 protein complex content, as suggested by Hagiwara et al. [74] and Yuan et al. [75]. After 48 h, the use of resveratrol, *cis-9*, *trans-11* CLA, and *trans-10*, *cis-12* CLA significantly increased the expression of *Prkaa1* and *Prkaa2* (Table 2). This confirmed that AMPK effectively maintains intensified ACC and SREBP control even under oxidative stress conditions.

This study found that the combination of resveratrol with *trans-10*, *cis-12* CLA had a stronger inhibitory effect on malonyl-CoA synthesis and changes in AMPK-related gene expression compared to the combination of resveratrol, *cis-9*, *trans-11* CLA, and *trans-10*, *cis-12* CLA (Figure 4, Table 2). The findings indicated that the reduction in de novo fatty acid biosynthesis intensity in cells subjected to oxidative stress, which was attributed to a decline in ACC and FASN protein levels, was less pronounced than under standard culture conditions (Figures S8–S14). Notably, there was a decrease in *Acc1* and *Fasn* gene expression after 48 h (Figure 4) under oxidative stress conditions, which was not observed under standard culture conditions.

## 3. Materials and Methods

### 3.1. Chemical Reagents

Cell culture reagents: Dulbecco’s Modified Eagle’s Medium (Sigma Aldrich, St. Louis, MI, USA, cat no D6429); Fetal bovine serum (Sigma Aldrich, St. Louis, MI, USA, cat no F4135); Penicillin/streptomycin antibiotic mixture (Sigma Aldrich, St. Louis, MI, USA, cat no P4458); PBS (Sigma Aldrich, St. Louis, MI, USA, cat no P4474); Trypsin-EDTA solution (Sigma Aldrich, St. Louis, MI, USA, cat no T4049); Isobutylmethylxanthine IBMX (Sigma Aldrich, St. Louis, MI, USA, cat no I5879); dexamethasone DEX (Sigma Aldrich, St. Louis, MI, USA, cat no D4902); Bovine insulin (Sigma Aldrich, St. Louis, MI, USA, cat no I6634); Resveratrol trans-3,4ʹ,5-trihydroxystilbene (Sigma-Aldrich St. Louis, MI, USA, cat no 554325); the *cis-9*, *trans-11* CLA isomer (Sigma-Aldrich St. Louis, MI, USA, cat no 16413); the *trans-10*, *cis-12* CLA isomer (Sigma-Aldrich St. Louis, MI, USA, cat no 04397); 2,2′-Azobis (Sigma-Aldrich St. Louis, MI, USA, cat no 11596); Triton X-100 (Sigma-Aldrich St. Louis, MI, USA, cat no T8787).

Reagent kits: Cytotoxicity Detection Kit LDH (Roche, Mannheirm, Germany, cat no 11644793001); Total RNA Mini Plus Kit (A&A Biotechnology, Gdynia, Poland, cat no 036–100); iScript Reverse Transcription Supermix (Bio-Rad, Hercules, CA, USA, cat no 1708840); SYBR^®^ Green (SooAdvanced Universal SYBR^®^Green Supermix, BioRad, Hercules, CA, USA, cat no 1725271) Pierce™ BCA Protein Assay Kit (Thermo Fisher Scientific, Waltham, MA, USA, cat no A55860); Fatty Acid and Lipid Metabolism Antibody Sampler Kit (Cell Signaling Technology, Danvers, MA, USA, cat no 8335).

Other reagents: DMSO (Sigma-Aldrich St. Louis, MI, USA, cat no D8418); Water for molecular biology (Sigma-Aldrich St. Louis, MI, USA, cat no 693520); RNase away (Sigma-Aldrich St. Louis, MI, USA, cat no 83931); Cell Lysis Buffer RIPA (Sigma-Aldrich St. Louis, MI, USA, cat no 20–188); Protease Inhibitor Cocktail (BioShop, ON, Canada, cat no PIC002.1); Mini-PROTEAN TGX Stain-Free precast gels (Bio-Rad, Hercules, CA, USA, cat no 4568024); Trans-Blot Turbo Mini Nitrocellulose Transfer Packs (Bio-Rad, Hercules, CA, USA, cat no 1704158); Glycine (Sigma-Aldrich St. Louis, MI, USA, cat no G7126); Trizma^®^ (Sigma-Aldrich St. Louis, MI, USA, cat no T1503); Tween-20 (Sigma-Aldrich St. Louis, MI, USA, cat no 655206); Laemmli Sample Loading Buffer (Sigma-Aldrich St. Louis, MI, USA, cat no G2526); Protein size standard (Eurex, Gdańsk, Poand, cat no E3210); ClarityTM Western ECL Substrate (Bio-Rad, Hercules, CA, USA, cat no 1705061). 

### 3.2. Cell Culture

This research was conducted using the mouse fibroblast cell line 3T3-L1 (ATCC^®^ CL-173™), purchased from the American Type Culture Collections (ATCC, Manassas, VA, USA). The cells were cultured under controlled conditions (atmosphere: air 95%; CO_2_ 5%; temperature 37 °C) in an incubator (NuAire, Plymouth, MN, USA) and in an appropriate medium with the addition of 10% fetal bovine serum (FBS, Life Technologies, Carlsbad, CA, USA), supplements, and an antibiotic mixture penicillin/streptomycin (Life Technologies, Carlsbad, CA, USA) following the ATCC protocol. The culture medium was refreshed every 2–3 days, and passage was carried out at 50–60% confluence. 

### 3.3. Differentiation into Adipocytes

The differentiation of 3T3-L1 fibroblasts into adipocytes was confidently performed on either 6- or 96-well plates coated with collagen (Sigma Aldrich, St. Louis, MI, USA) to ensure optimal adhesion to the substrate. For the cytotoxicity test, 96-well plates were used, while 6-well plates were used for each experiment. Only cells between 8 and 15 passages were selected for the differentiation procedure. The collagen-coated plates were expertly seeded with either 4 × 10^3^ cells per well (96-well plate) or 8 × 10^4^ cells per well (6-well plate, Sarstedt, Numbrecht, Germany). The 3T3-L1 fibroblasts were seeded and cultured until full confluence was reached in the growth medium. The medium was then replaced with fresh medium, and the cells were left in the incubator for an additional 48 h. Differentiation was initiated 4 days after seeding the cells on plates by changing the growth medium to a differentiation medium. The DMEM used in this study contained 4.5 g/L of high glucose and was supplemented with L-glutamine, sodium pyruvate, and sodium bicarbonate (Sigma Aldrich, St. Louis, MI, USA). Additionally, it was enriched with 10% FBS and a mixture of antibiotics. The cells received treatment with 5 mM isobutylmethylxanthine (IBMX, Sigma Aldrich, St. Louis, MI, USA), 1 µM dexamethasone (DEX, Sigma Aldrich, St. Louis, MI, USA), and 1 µg/mL bovine insulin (Sigma Aldrich, St. Louis, MI, USA). After 72 h, the medium was replaced by a differentiation medium with insulin. The insulin medium was composed of DMEM with high glucose content [4.5 g/L], L-glutamine, sodium pyruvate, and sodium bicarbonate (Sigma Aldrich, St. Louis, MI, USA). The 3T3-L1 preadipocytes were incubated in a medium containing 10% FBS, penicillin/streptomycin antibiotic mixture, and 1 µg/mL bovine insulin (Sigma Aldrich, St. Louis, MI, USA). Over 90% of the cells developed visible lipid drops and were ready for testing after 11 to 18 days of differentiation. The tested substances were added to 3T3-L1 adipocytes on the 12th day of differentiation.

### 3.4. Cell Treatment

To determine whether the tested substances retained similar properties under conditions of oxidative stress, 3T3-L1 fibroblasts were differentiated into mature adipocytes. The cells were then treated with resveratrol (trans-3,4′,5-trihydroxystilbene; cat no 554325; Sigma-Aldrich St. Louis, MI, USA), the *cis-9*, *trans-11* CLA isomer (cat no 16413; Sigma-Aldrich St. Louis, MI, USA), or the *trans-10*, *cis-12* CLA isomer (cat no 04397; Sigma-Aldrich St. Louis, MI, USA). These substances were added in all possible combinations, including resveratrol [75 µM], *cis-9*, *trans-11* CLA [50 µM], *trans-10*, *cis-12* CLA [50 µM] and a combination of *cis-9*, *trans-11* CLA [50 µM] + *trans-10*, *cis-12* CLA [50 µM]. This study investigated the effects of different combinations of resveratrol and CLA on adipose tissue processes in obese individuals under oxidative stress conditions induced by 2,2′-Azobis (AAPH) [1.5 mM]. The negative control for all experiments consisted of untreated cells (UC) grown in complete culture medium without any materials. To solubilize membrane proteins in the LDH assay, Triton X-100 [2%] (Sigma Aldrich, St Louis, MI, USA) was used as a positive control. 

### 3.5. Cytotoxicity Assay

Cells were seeded into 96-well plates at 4 × 10^3^ cells/well 24 h before the planned experiment. Cytotoxicity was assessed after 24, 48, and 72 h incubation of the cells with the analyzed substances using the Cytotoxicity Detection Kit LDH (Roche, Warsaw, Poland). After the incubation time, the medium was transferred to a new 96-well plate. To the collected medium, 100 μL of an ex tempore prepared reaction mixture containing lactic acid, NAD+, tetrazolium salt, and diaphorase was added. The plates were incubated for 30 min at room temperature without light. The intensity of the staining produced during this time was directly proportional to the degree of cell damage and was measured spectrophotometrically at 490 nm using a Multiscan GO microplate reader (Thermo Scientific, Waltham, MA, USA). The negative control was a culture of cells treated with no substances, while the positive control was a culture supplemented with 2% Triton X-100 (Sigma-Aldrich, USA), which caused lysis of 100% of the cells. 

### 3.6. RNA Isolation, cDNA Synthesis, and RT-qPCR 

For the RNA, isolation cells were seeded in 6-well plates at 8 × 10^4^ and treated as was described above in Section 3.2. RNA isolation from cell cultures was performed using a Total RNA Mini Plus Kit (A&A Biotechnology, Gdynia, Poland) according to the manufacturer’s instructions. The concentration, purity, and quality of the isolated RNA were measured using UV-Vis NanoDrop 2000 (Thermofisher, Waltham, MA, USA). Subsequently, cDNA was synthesized using iScript Reverse Transcription Supermix (Bio-Rad, Hercules, CA, USA) via a reverse transcriptase (RT) reaction. Gene expression was quantitatively verified using the QuantStudio 12K Flex Real-Time PCR System. 

### 3.7. Western Blot Assays

Cell lysis was performed with Cell Lysis Buffer RIPA (Sigma Aldrich, St. Louis, Missouri, USA). The protein content in the cell lysates was quantified using the Pierce™ BCA Protein Assay Kit (Thermo Fisher Scientific, Waltham, MA, USA) and the Multiscan GO Microplate Reader (Thermo Fisher Scientific, Waltham, MA, USA). Cell lysate proteins were separated using Mini-PROTEAN TGX Stain-Free precast gels (Bio-Rad, Hercules, CA, USA). The Trans-Blot Turbo Mini Nitrocellulose Transfer Packs (Bio-Rad, Hercules, CA, USA) were used with the Trans-Blot^®^ Turbo^TM^ Transfer System (Bio-Rad, Hercules, CA, USA) to ensure optimal protein transfer from gels. The immobilized proteins were then incubated with the appropriate primary antibody (ACC, FAS,) to complete the process. The appropriate secondary antibody conjugated with horseradish peroxidase (Cell Signaling Technology, Danvers, MA, USA.) was applied, followed by detection using Clarity^TM^ Western ECL Substrate (Bio-Rad, Hercules, CA, USA) and visualization with the Chemi-Doc^TM^ Imaging System (Bio-Rad, Hercules, CA, USA). Densitometric assays were performed using ImageLab (by author Wayne Rasband). 

### 3.8. Statistical Analysis

Each experiment was performed independently six times. For the gene and protein expression, the experiments were polled. The results are presented as the mean of three repetitions ± standard deviation (SD). Results with a *p*-value of less than 0.01 were denoted with two asterisks (**), while those with a *p*-value of less than 0.001 were denoted with three asterisks (***). Statistical significance between two groups was determined using the T test (Prism, GraphPad Software, Boston, MA, USA). Results with a *p*-value of less than 0.05 were considered statistically significant and denoted with an asterisk (*). Results with a *p*-value of less than 0.0001 were denoted with four asterisks (****).

## 4. Conclusions

This study investigated the impact of resveratrol, *cis-9*, *trans-11* CLA, and *trans-10*, *cis-12* CLA isomers both individually and in combination on de novo fatty acid biosynthesis in 3T3-L1 adipocytes under standard and oxidative stress conditions. Resveratrol showed significant inhibitory effects on fatty acid biosynthesis, particularly evident in reduced ACC and FASN protein levels. Similarly, both CLA isomers, when applied alone, exhibit inhibitory effects on fatty acid biosynthesis pathways. However, the mixture of resveratrol and *trans-10*, *cis-12* CLA proved to be more effective in reducing malonyl-CoA synthesis and downregulated FASN expression compared to individual components.

Under oxidative stress, these compounds maintained their inhibitory effects on fatty acid biosynthesis, although they were less effective. The combination of resveratrol, *cis-9*, *trans-11 CLA*, and *trans-10*, *cis-12* CLA showed results in reducing fatty acid synthesis pathways, as indicated by decreased ACC and FASN protein levels and alterations in gene expression related to lipid metabolism.

This study underscores the importance of ingredient combinations in modulating cellular responses and highlights potential avenues for controlling lipid metabolism. Further research into the underlying molecular mechanisms and long-term effects of these combinations is crucial. The use of 3T3-L1 adipocytes, as a standard model for adipogenesis and lipid metabolism, may not fully represent the complexity of human adipose tissue in vivo. Although this study observed changes in protein levels and gene expression related to fatty acid biosynthesis, the precise molecular mechanisms underlying the observed effects of resveratrol and CLA isomers, both individually and in combination, were not fully elucidated. Detailed studies, including signaling pathways and metabolic analyses, would provide better understanding.

## Figures and Tables

**Table 1:** Expression of selected genes related to fatty acid biosynthesis in 3T3-L1 adipocytes treated with resveratrol (75 µM), the *trans-10*, *cis-12* CLA isomer (50 µM), and with a mixture of *cis-9*, *trans-11* CLA (50 µM) and *trans-10*, *cis-12* CLA (50 µM) under standard conditions and with induced oxidative stress (os), after 24 and 48 h of incubation.

resveratrol									
expression (% of control) ^1^	gene	standard conditions				oxidative stress			
		24 h		48 h		24 h		48 h	
		level	*p*	level	*P*	level	*p*	level	*p*
	*Acly*	84.4 ± 1.8	****	118.2 ± 1.8	****	121.9 ± 2.6	****	76.6 ± 1.7	****
	*Srebp1*	49.6 ± 1.8	****	128.0 ± 7.4	****	69.5 ± 0.6	****	96.1 ± 3.7	ns
	*Prkaa1*	93.9 ± 3.2	ns	174.4 ± 4.6	****	153.6 ± 4.4	****	140.9 ± 4.2	****
	*Prkaa2*	95.2 ± 7.4	ns	102.2 ± 9.6	Ns	168.2 ± 8.8	****	83.3 ± 9.2	*
	*Prkaca*	63.4 ± 1.3	****	143.3 ± 3.3	****	101.2 ± 2.1	ns	122.1 ± 0.6	**
*cis-9*, *trans-11* CLA									
expression (% of control) ^1^		standard conditions				oxidative stress			
		24 h		48 h		24 h		48 h	
		level	*p*	level	*P*	level	*p*	level	*p*
	*Acly*	85.0 ± 12.2	*	102.7 ± 2.3	Ns	124,3 ± 1.8	**	85.4 ± 4.8	ns
	*Srebp1*	50.5 ± 6.3	****	106.8 ± 2.1	Ns	82.9 ± 3.8	**	84.1 ± 1.6	**
	*Prkaa1*	106.6 ± 5.9	ns	108.2 ± 2.3	*	108.6 ± 1.4	**	103.7 ± 3.0	ns
	*Prkaa2*	68.1 ± 1.9	****	99.7 ± 6.5	Ns	96.7 ± 3.9	ns	104.6 ± 4.2	ns
	*Prkaca*	65.3 ± 4.6	****	114.2 ± 2.1	***	112.2 ± 3.7	***	91.6 ± 1.2	**
*trans-10*, *cis-12* CLA									
expression (% of control) ^1^		standard conditions				oxidative stress			
		24 h		48 h		24 h		48 h	
		level	*p*	level	*P*	level	*p*	level	*p*
	*Acly*	84.5 ± 0.6	***	125.8 ± 5.4	****	112.8 ± 4.8	ns	56.7 ± 2.2	***
	*Srebp1*	50.5 ± 1.0	****	114.4 ± 1.4	****	87.4 ± 5.1	*	62.7 ± 2.0	****
	*Prkaa1*	100.1 ± 2.5	ns	105.8 ± 1.9	*	118.0 ± 2.5	****	97.0 ± 1.8	ns
	*Prkaa2*	62.6 ± 6.4	****	102.0 ± 2.0	Ns	122.2 ± 8.5	**	83.7 ± 4.5	*
	*Prkaca*	71.4 ± 1.1	****	111.5 ± 3.6	***	105.8 ± 2.0	*	73.3 ± 0.2	****
*cis-9*, *trans-11 CLA + trans-10*, *cis-12* CLA									
expression (% of control)^1^		standard conditions				oxidative stress			
		24 h		48 h		24 h		48 h	
		level	*p*	Level	*P*	level	*p*	level	*p*
	*Acly*	110.0 ± 0.8	**	113.9 ± 3.2	***	130.8 ± 3.0	**	70.6 ± 0.2	**
	*Srebp1*	67.0 ± 2.4	****	112.6 ± 2.5	**	88.3 ± 1.6	*	73.4 ± 2.8	***
	*Prkaa1*	97.1 ± 2.5	ns	103.3 ± 1.3	Ns	117.4 ± 1.1	****	65.4 ± 1.3	**
	*Prkaa2*	83.5 ± 0.2	***	98.9 ± 3.0	Ns	125.5 ± 2.7	***	88.1 ± 5.5	*
	*Prkaca*	89.6 ± 0.8	***	114.6 ± 1.8	****	114.8 ± 3.2	***	82.7 ± 2.2	****

^1^ Gene expression values are presented in relation to those obtained in control cells, *n* = 3. The statistical significance of differences was determined by the T test at α = 0.05 (ns *p* > 0.05; * *p* < 0.05; ** *p* < 0.01; *** *p* < 0.001; **** *p* < 0.0001).

**Table 2:** Expression of selected genes related to fatty acid biosynthesis in 3T3-L1 adipocytes treated with a mixture of resveratrol (75 µM) and *cis-9*, *trans-11* CLA (50 µM), the *trans-10*, *cis-12* CLA isomer (50 µM), a mixture of resveratrol (75 µM) and *trans-10*, *cis-12* CLA (50 µM), and with a mixture of resveratrol (75 µM), cis*-9*, *trans-11* CLA (50 µM) and *trans-10*, *cis-12* CLA (50 µM) under standard conditions and with induced oxidative stress (os), after 24 and 48 h of incubation.

resveratrol + *cis-9*, *trans-11* CLA									
expression (% of control)^1^	gene	standard conditions				oxidative stress			
		24 h		48 h		24 h		48 h	
		level	*p*	level	*P*	level	*p*	level	*p*
	*Acly*	99.6 ± 0.1	ns	120.1 ± 1.4	****	121.6 ± 0.9	**	70.6 ± 3.4	***
	*Srebp1*	66.2 ± 10.9	***	143.2 ± 2.9	****	105.8 ± 5.2	ns	93.5 ± 5.5	ns
	*Prkaa1*	120.0 ± 2.0	**	152.5 ± 10.1	****	109.2 ± 0.6	***	112.4 ± 1.5	****
	*Prkaa2*	130.0 ± 4.4	***	101.4 ± 7.4	Ns	139.5 ± 6.5	****	77.7 ± 4.8	**
	*Prkaca*	74.1 ± 1.9	****	149.6 ± 3.2	****	93.6 ± 2.9	*	115.3 ± 0.6	****
resveratrol + *trans-10*, *cis-12* CLA									
expression (% of control) ^1^		standard conditions				oxidative stress			
		24 h		48 h		24 h		48 h	
		level	*p*	level	*P*	level	*p*	level	*p*
	*Acly*	144.6 ± 4.1	****	109.1 ± 4.6	*	101.8 ± 2.1	ns	97.1 ± 2.0	ns
	*Srebp1*	65.9 ± 1.0	****	128.1 ± 5.2	****	86.1 ± 3.0	*	111.2 ± 0.5	ns
	*Prkaa1*	157.8 ± 2.1	****	141.6 ± 1.7	****	117.6 ± 6.0	**	128.2 ± 5.0	****
	*Prkaa2*	204.7 ± 4.7	****	90.2 ± 9.0	Ns	169.5 ± 10.4	****	103.6 ± 4.7	ns
	*Prkaca*	84.6 ± 1.6	***	134.9 ± 3.3	****	87.3 ± 1.8	***	137.9 ± 1.6	****
resveratrol *+ cis-9*, *trans-11* CLA + *trans-10*, *cis-12* CLA									
expression (% of control) ^1^		standard conditions				oxidative stress			
		24 h		48 h		24 h		48 h	
		level	*p*	level	*P*	level	*p*	level	*p*
	*Acly*	131.0 ± 3.6	****	130.4 ± 3.2	****	112.8 ± 3.3	*	55.4 ± 3.9	****
	*Srebp1*	50.0 ± 3.3	****	155.3 ± 7.7	****	96.6 ± 4.2	ns	70.2 ± 2.5	**
	*Prkaa1*	173.5 ± 4.9	****	179.0 ± 3.9	****	104.5 ± 0.6	ns	170.9 ± 2.9	****
	*Prkaa2*	213.8 ± 3.5	****	114.6 ± 5.4	*	139.2 ± 4.2	****	110.9 ± 5.6	*
	*Prkaca*	94.6 ± 0.1	*	158.4 ± 3.1	****	98.8 ± 14.0	ns	113.8 ± 10.3	*

^1^ Gene expression values are presented in relation to those obtained in control cells (*n* = 3). The statistical significance of differences was determined by the T test at α = 0.05 (ns *p* > 0.05; * *p* < 0.05; ** *p* < 0.01; *** *p* < 0.001; **** *p* < 0.0001).

## Data Availability

Data will be made available on request.

## Supplementary Materials

The following supporting information can be downloaded at https://www.mdpi.com/article/10.3390/ijms252413429/s1.
