# Supplementary material for: The Combination of Resveratrol and Conjugated Linoleic Acid Dienes Enhances the Individual Effects of These Molecules on De Novo Fatty Acid Biosynthesis in 3T3-L1 Adipocytes
Source: Int J Mol Sci. 2024 Dec 14;25(24):13429. doi: 10.3390/ijms252413429 (PMC11677705; doi:10.3390/ijms252413429)
Supplement: Supplementary file 1 [file ijms-25-13429-s001.zip › ijms-3294107-supplementary.pdf]

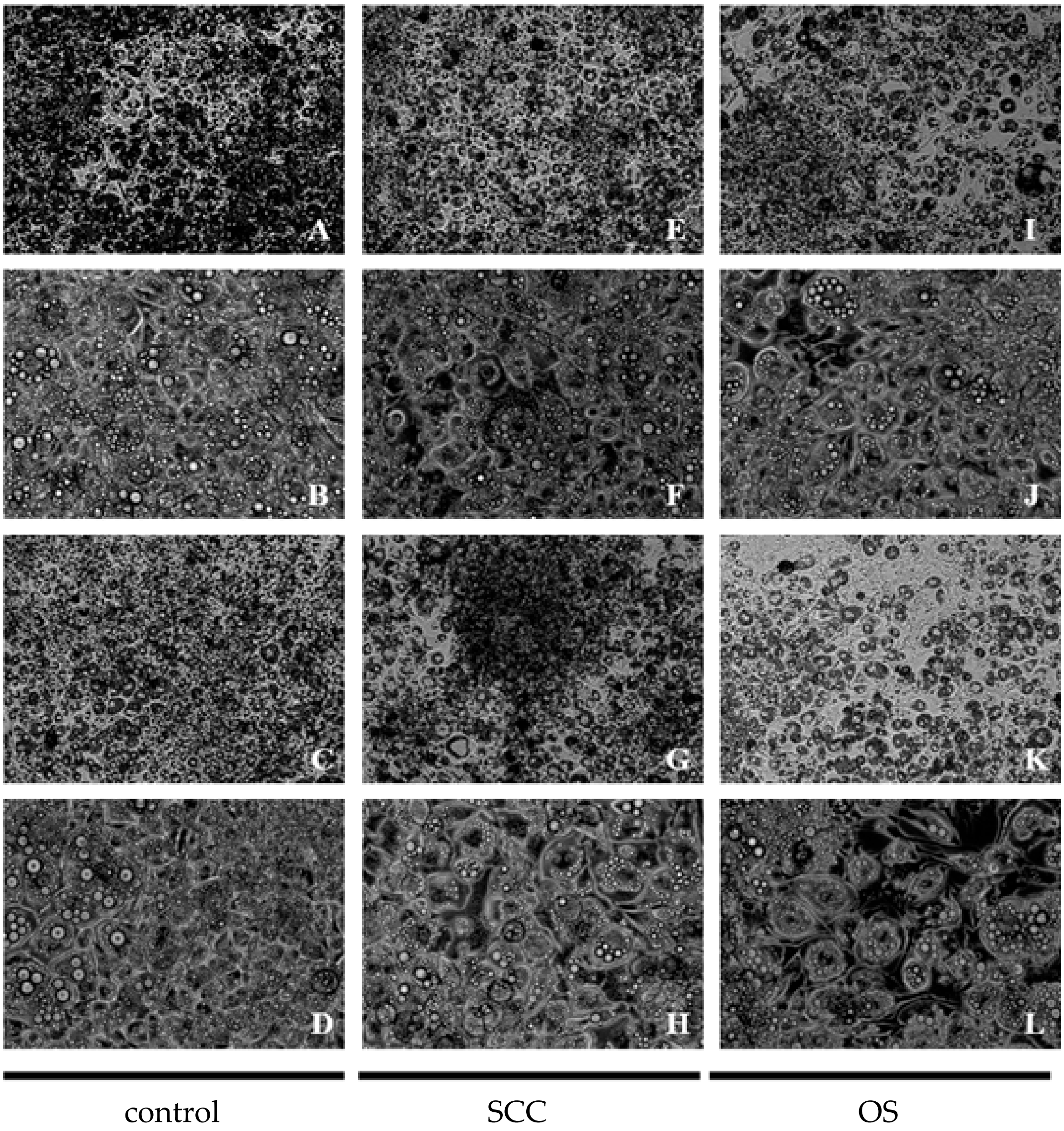

Figure S1. Morphology of 3T3-L1 adipocytes treated with resveratrol (75 μM) under standard culture conditions (SCC) and induced oxidative stress (OS). A, B, E, F, I, J - after 24 h of incubation. C, D, G, H, K, L - after 48 h of incubation (fot. Jarosław Oczkowicz).

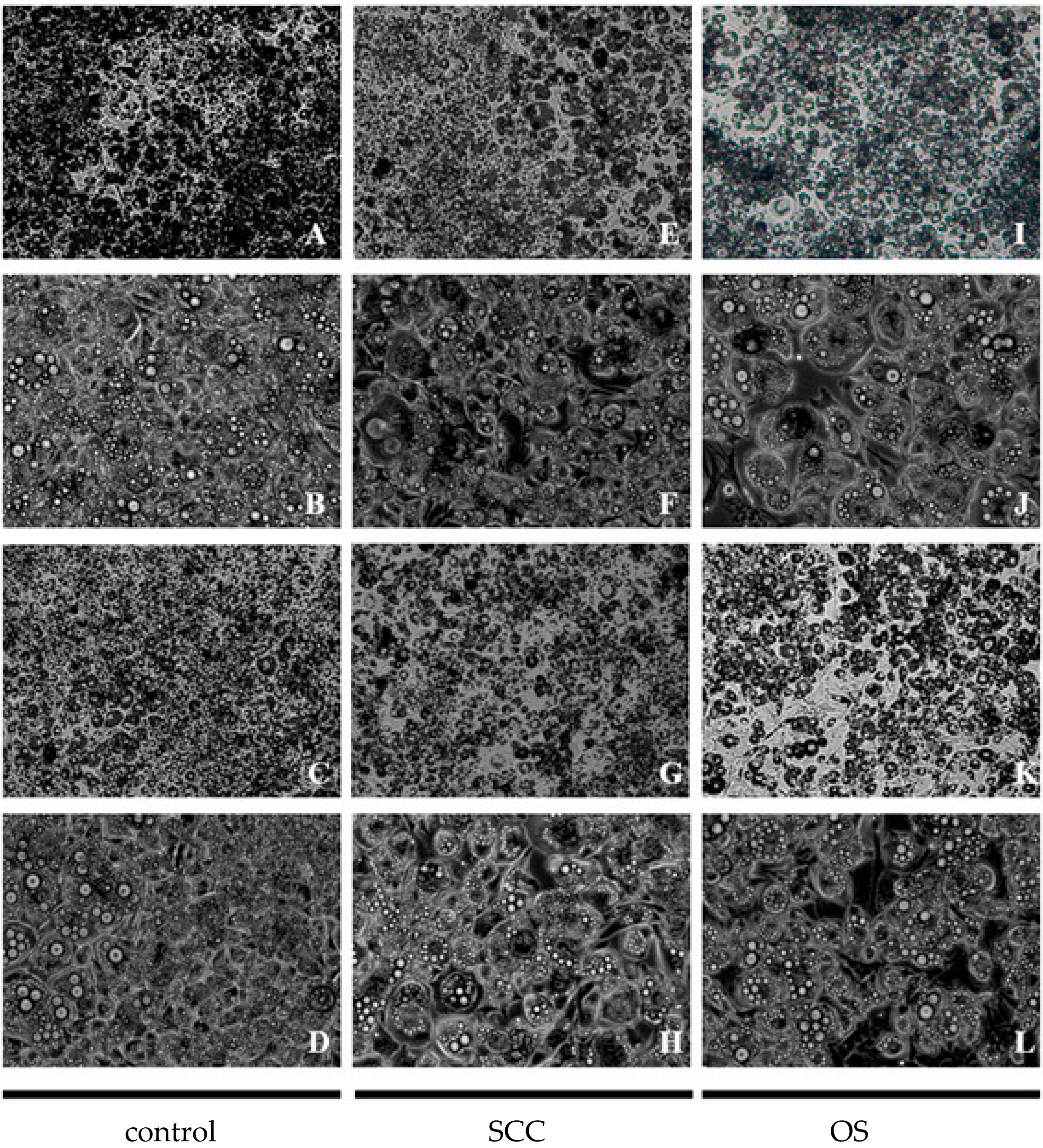

Figure S2. Morphology of 3T3-L1 adipocytes treated with *cis-9, trans-11* CLA (50 μM) under standard culture conditions (SCC) and induced oxidative stress (OS). A, B, E, F, I, J - after 24 h of incubation. C, D, G, H, K, L - after 48 h of incubation (fot. Jarosław Oczkowicz).

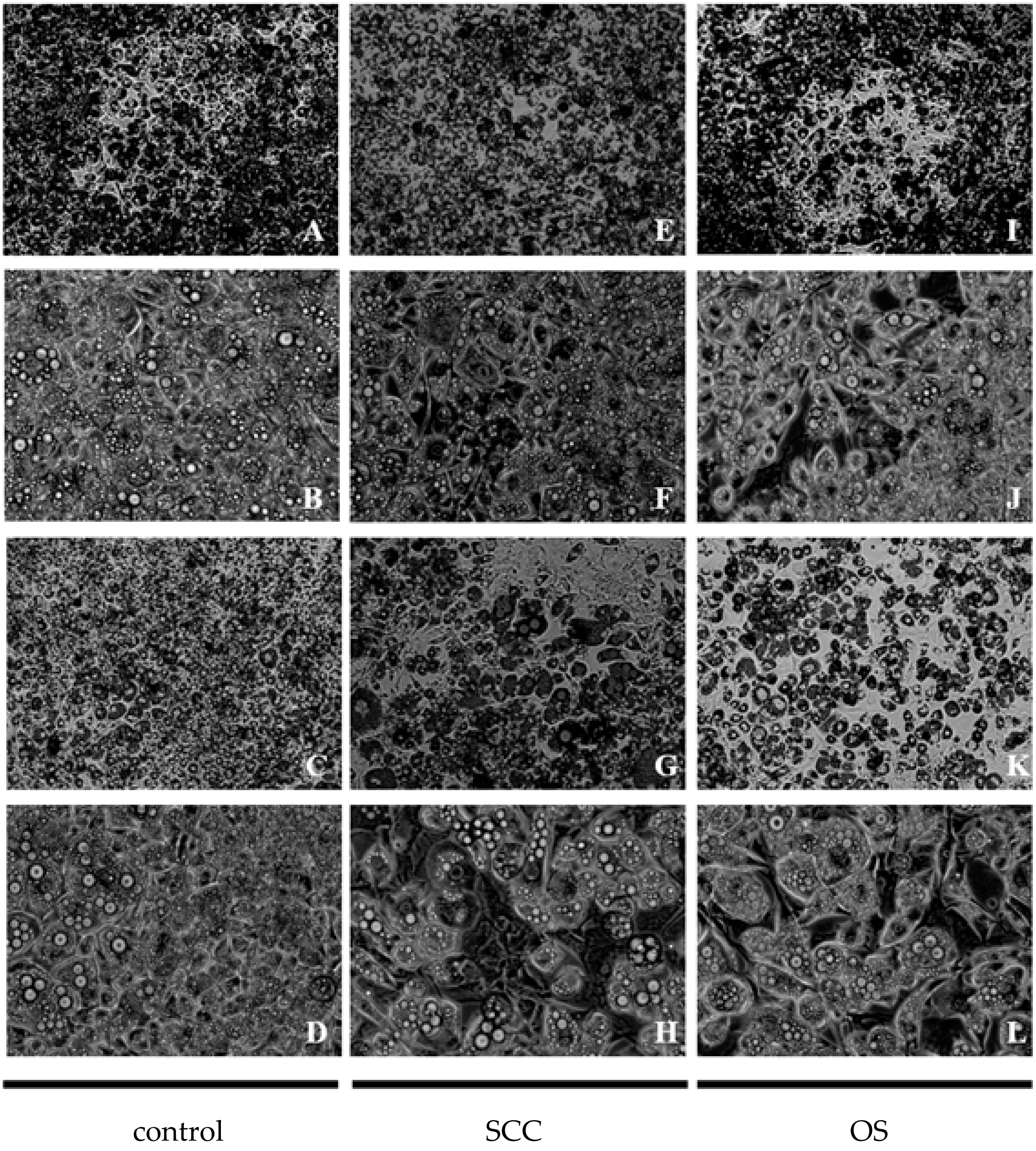

Figure S3. Morphology of 3T3-L1 adipocytes treated with *trans-10, cis-12* CLA (50 μM), under standard culture conditions (SCC) and induced oxidative stress (OS). A, B, E, F, I, J - after 24 h of incubation. C, D, G, H, K, L- after 48 h of incubation (fot. Jarosław Oczkowicz).

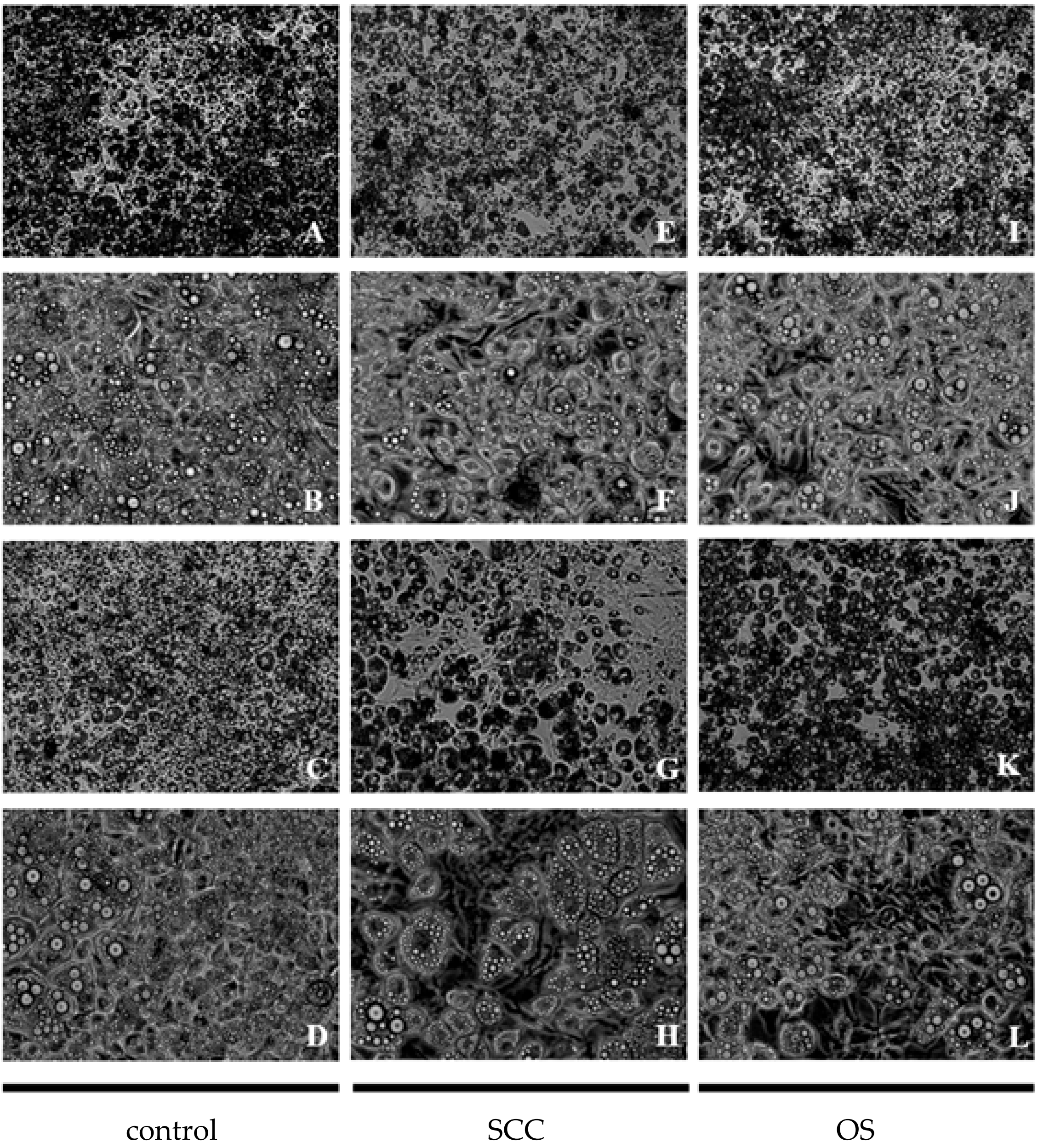

Figure S4. Morphology of 3T3-L1 adipocytes treated with a mixture of *cis-9, trans-11 CLA* (50 μM*)* and *trans-10, cis-12* CLA (50 μM), under standard culture conditions (SCC) and induced oxidative stress (OS). A, B, E, F, I, J - after 24 h of incubation. C, D, G, H, K, L - after 48 h of incubation (fot. Jarosław Oczkowicz).

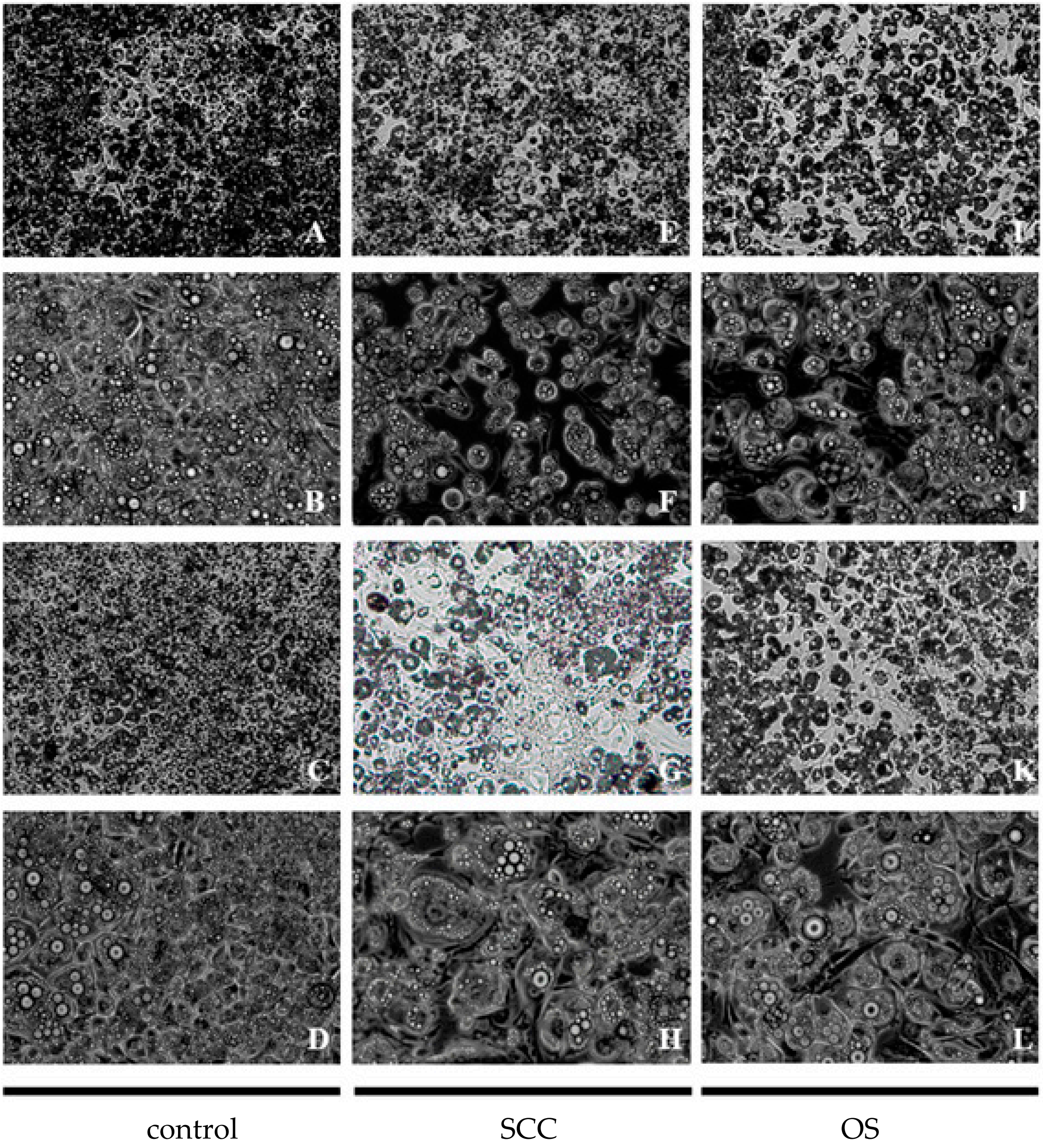

Figure S5. Morphology of 3T3-L1 adipocytes treated with a mixture of resveratrol (75 μM) and *cis-9*, *trans-11* CLA (50 μM), under standard culture conditions (SCC) and induced oxidative stress (OS). A, B, E, F, I, J - after 24 h of incubation. C, D, G, H, K, L - after 48 h of incubation (fot. Jarosław Oczkowicz).

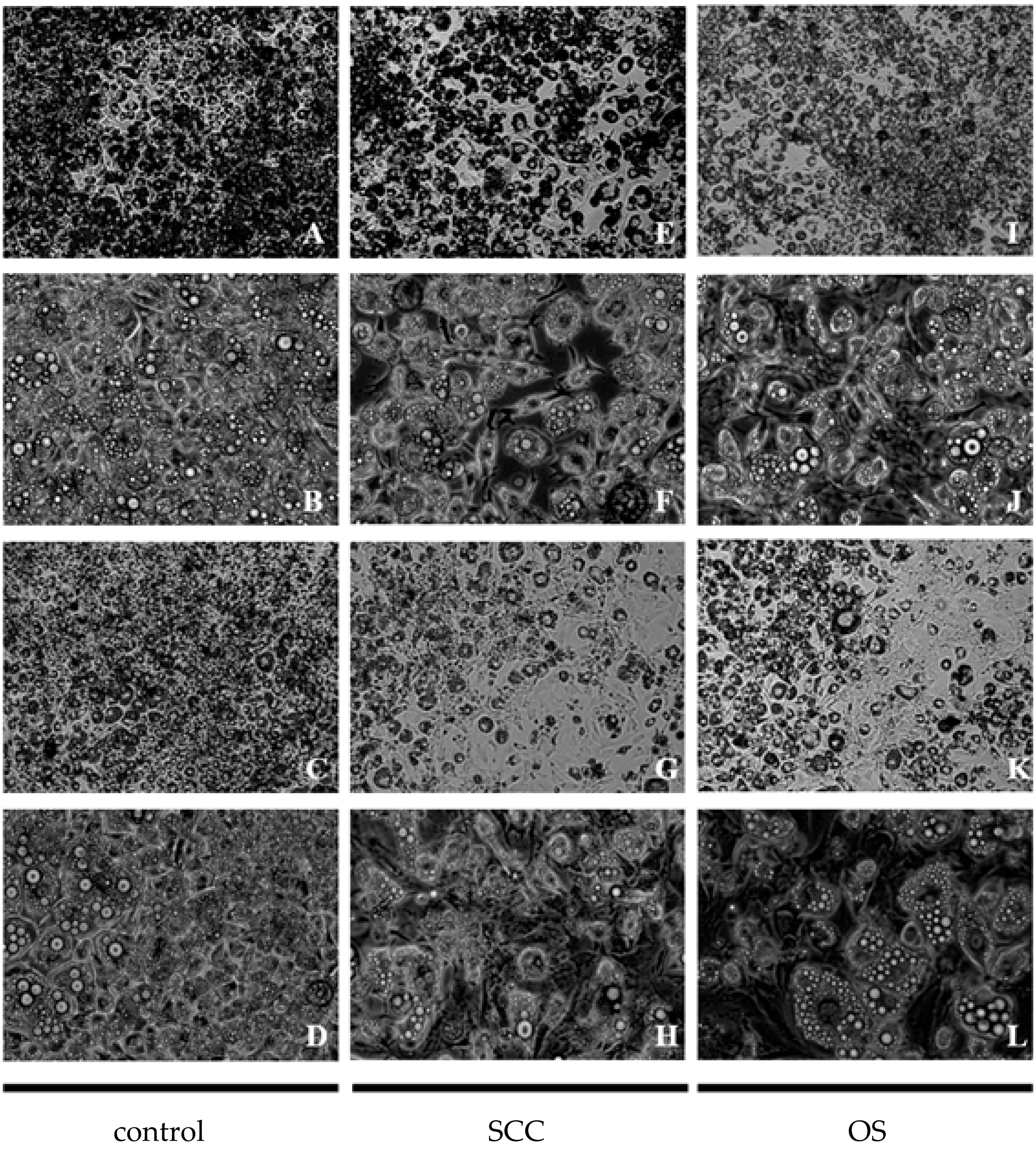

Figure S6. Morphology of 3T3-L1 adipocytes treated with a mixture of resveratrol (75 μM) and *trans-10, cis-12* CLA (50 μM), under standard culture conditions (SCC) and induced oxidative stress (OS). A, B, E, F, I, J - after 24 h of incubation. C, D, G, H, K, L - after 48 h of incubation (fot. Jarosław Oczkowicz).

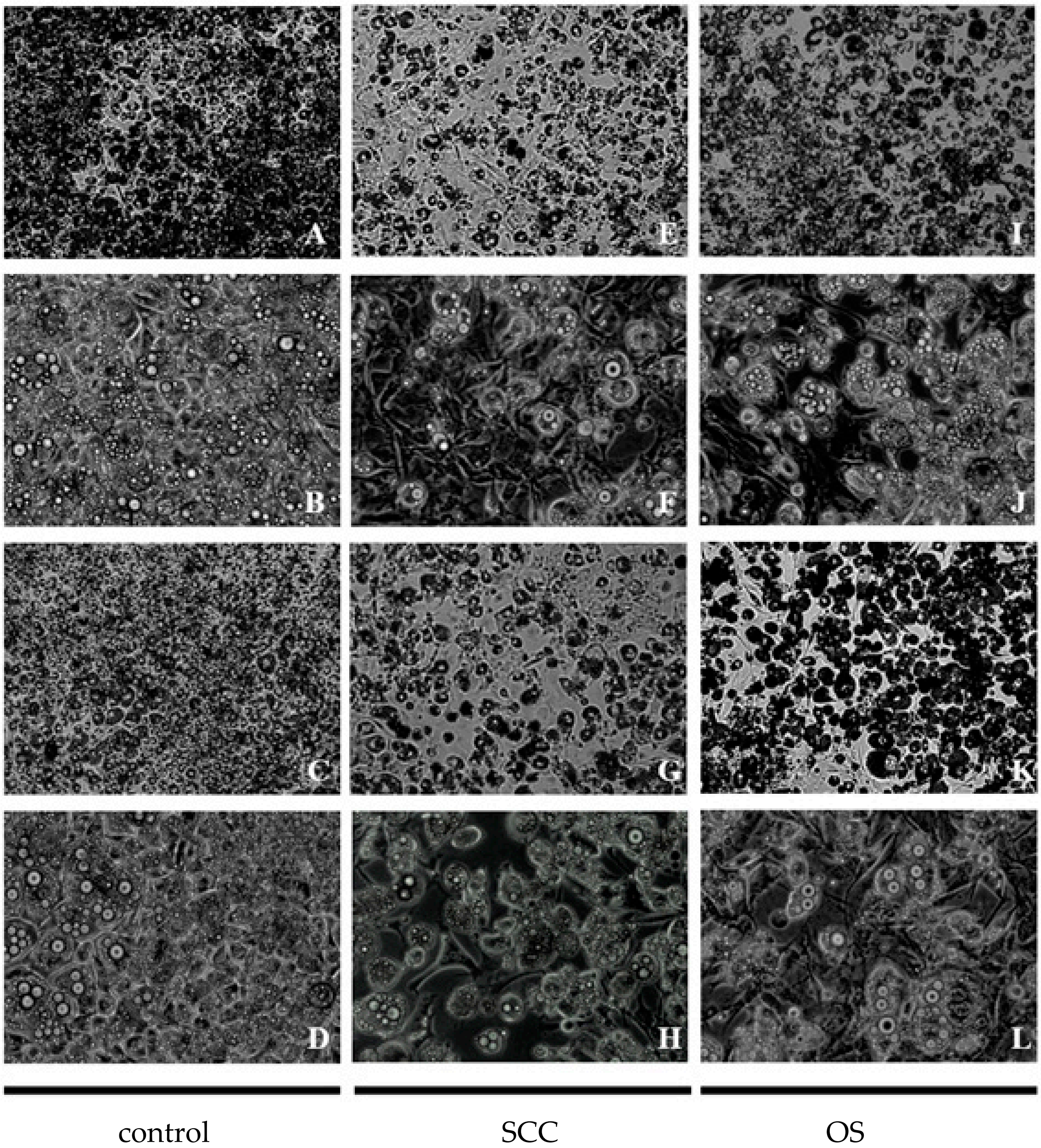

Figure S7. Morphology of 3T3-L1 adipocytes treated with a mixture of resveratrol (75 μM) *cis-9, trans-11* CLA and *trans-10, cis-12* CLA (50 μM), under standard culture conditions (SCC) and induced oxidative stress (OS). A, B, E, F, I, J - after 24 h of incubation. C, D, G, H, K, L - after 48 h of incubation (fot. Jarosław Oczkowicz).

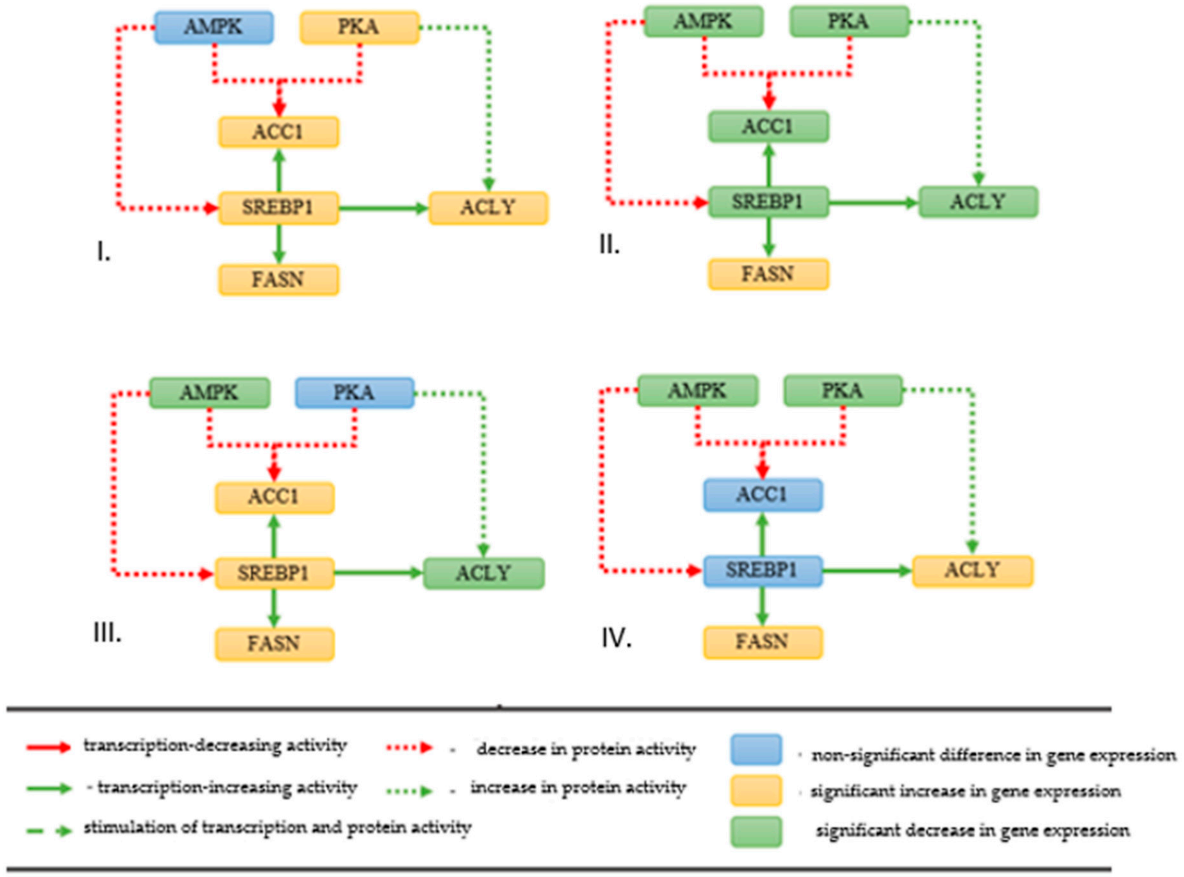

Figure S8. Interactions related to fatty acid metabolism in 3T3-L1 adipocytes treated with resveratrol under standard and oxidative stress conditions, based on the scientific literature presented and the results obtained in this study. **I**. Biosynthesis of fatty acids after 24 h under standard conditions. **II**. Biosynthesis of fatty acids after 48 h under standard conditions. **III**. Biosynthesis of fatty acids after 24 h under oxidative stress conditions. **IV.** Biosynthesis of fatty acids after 48 h under oxidative stress conditions.

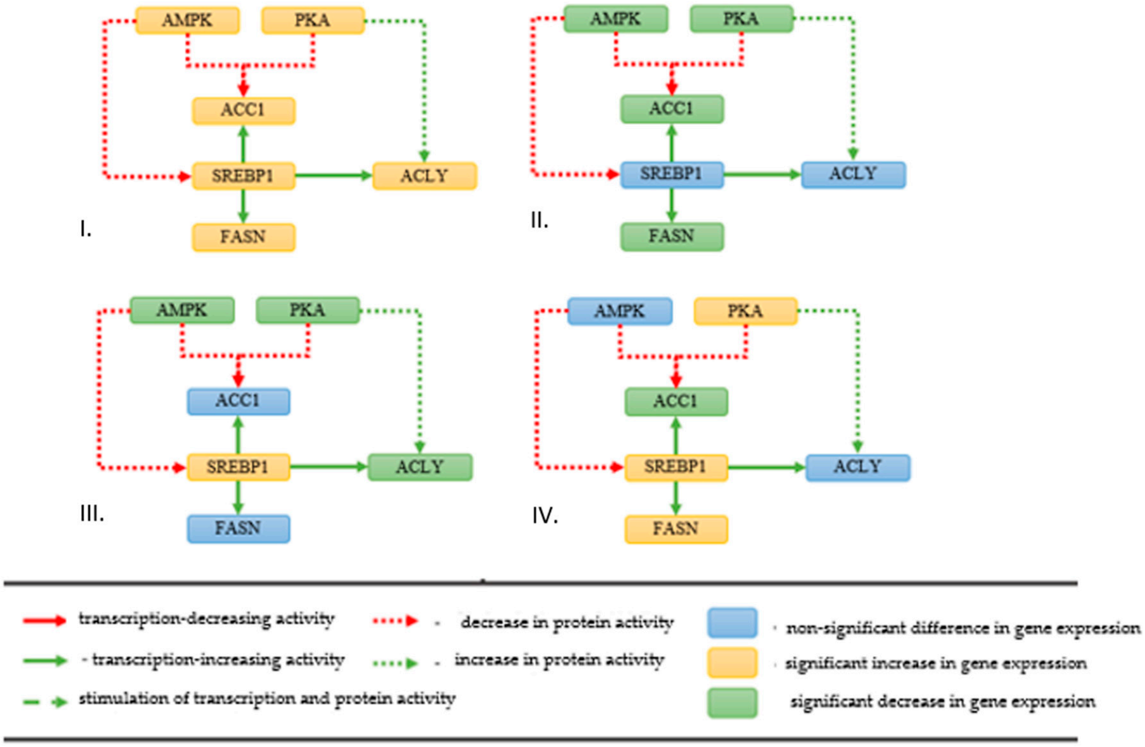

Figure S9. Interactions related to fatty acid metabolism in 3T3-L1 adipocytes treated with *cis-9,trans-11* CLA under standard and oxidative stress conditions, based on the scientific literature presented and the results obtained in this study. **I**. Biosynthesis of fatty acids after 24 h under standard conditions. **II**. Biosynthesis of fatty acids after 48 h under standard conditions. **III**. Biosynthesis of fatty acids after 24 h under oxidative stress conditions. **IV.** Biosynthesis of fatty acids after 48 h under oxidative stress conditions.

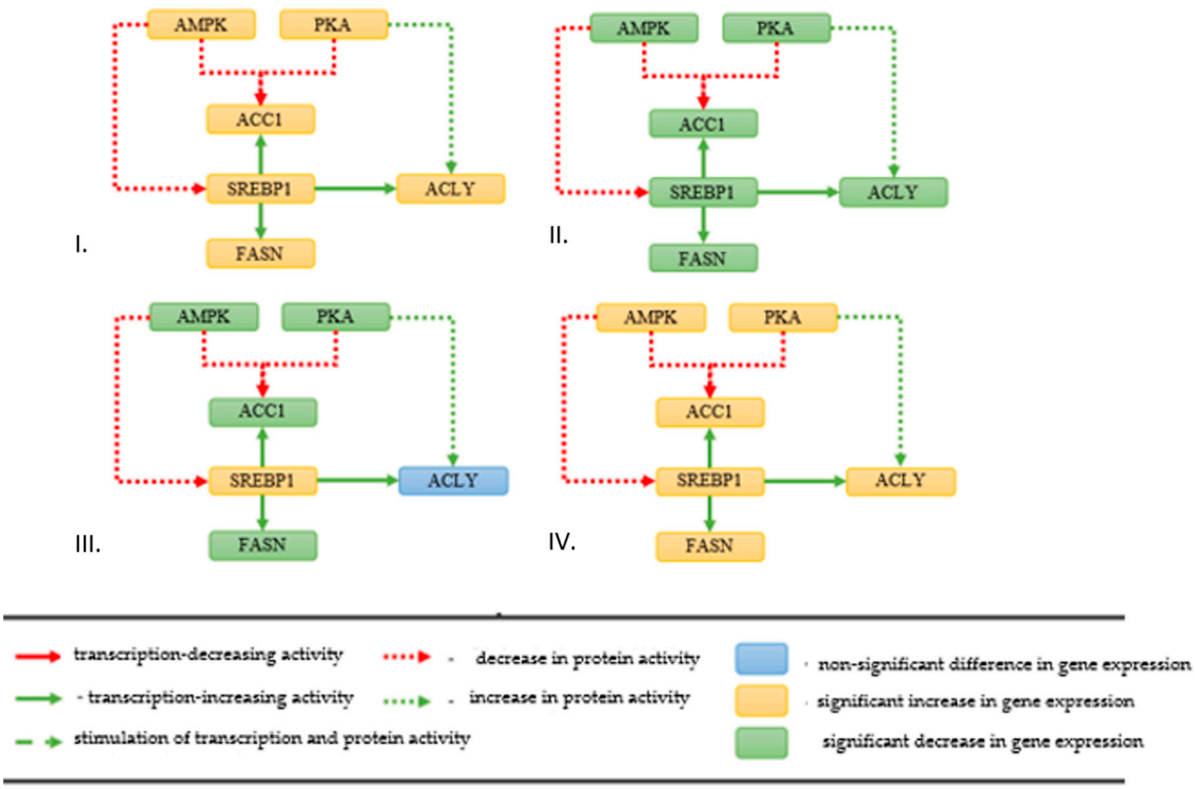

Figure S10. Interactions related to fatty acid metabolism in 3T3-L1 adipocytes treated with *trans-10,cis-11* CLA under standard and oxidative stress conditions, based on the scientific literature presented and the results obtained in this study. **I**. Biosynthesis of fatty acids after 24 h under standard conditions. **II**. Biosynthesis of fatty acids after 48 h under standard conditions. **III**. Biosynthesis of fatty acids after 24 h under oxidative stress conditions. **IV.** Biosynthesis of fatty acids after 48 h under oxidative stress conditions.

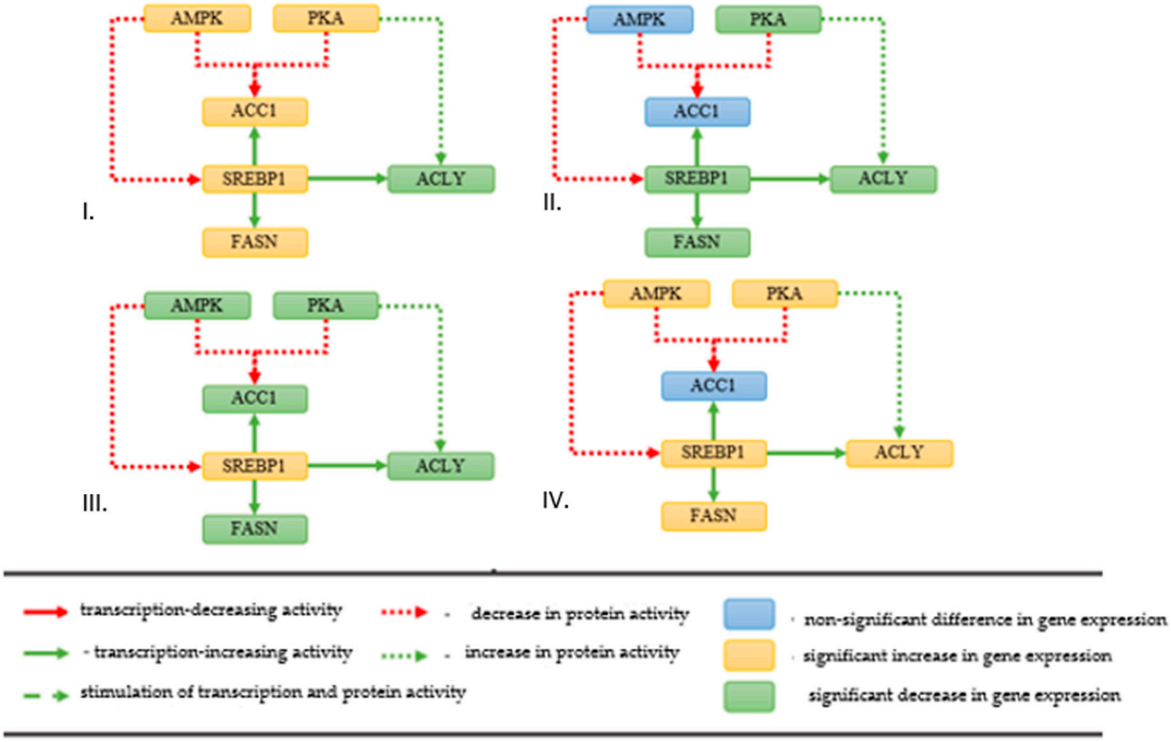

Figure S11. Interactions related to fatty acid metabolism in 3T3-L1 adipocytes treated with a mixture of *cis-9*, *trans-11* CLA (50 μM) and *trans-10, cis-12* CLA (50 μM) under standard and oxidative stress conditions, based on the scientific literature presented and the results obtained in this study. **I**. Biosynthesis of fatty acids after 24 h under standard conditions. **II**. Biosynthesis of fatty acids after 48 h under standard conditions. **III**. Biosynthesis of fatty acids after 24 h under oxidative stress conditions. **IV.** Biosynthesis of fatty acids after 48 h under oxidative stress conditions.

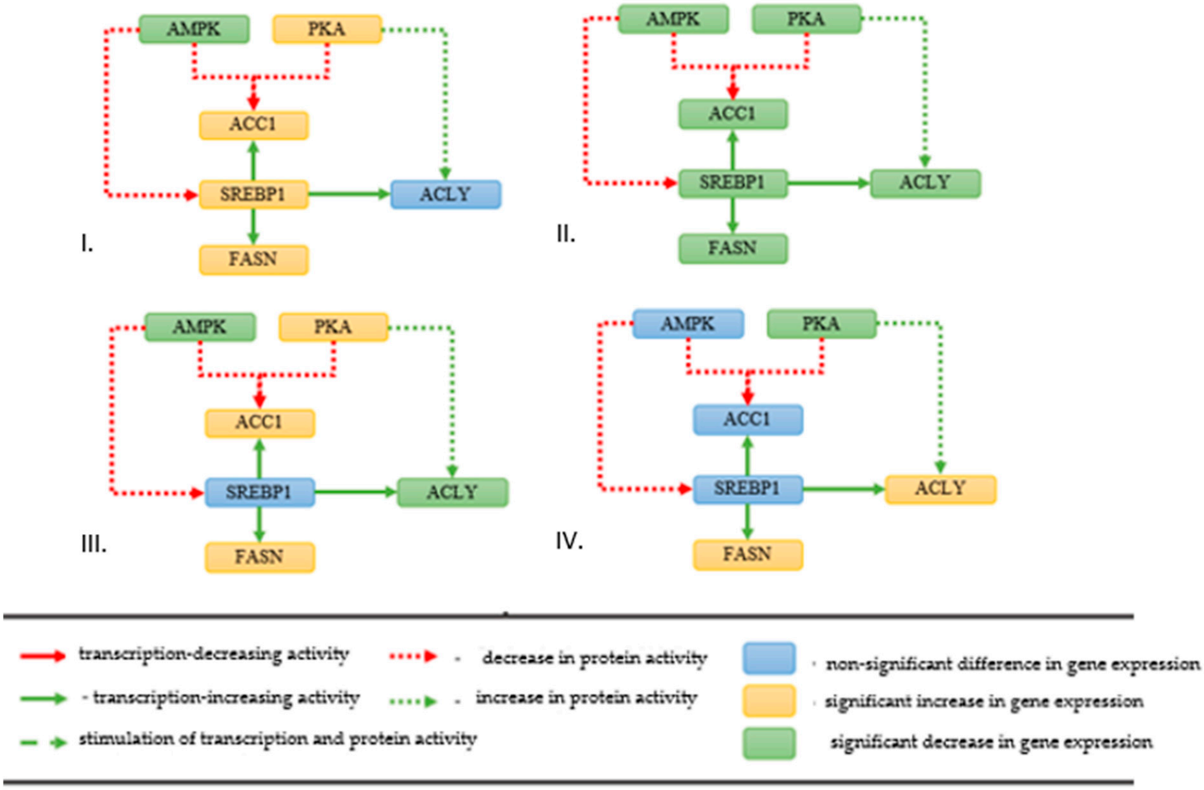

Figure S12. Interactions related to fatty acid metabolism in 3T3-L1 adipocytes treated with a mixture of a mixture of resveratrol (75 μM) and *cis-9, trans-11* CLA (50 μM) under standard and oxidative stress conditions, based on the scientific literature presented and the results obtained in this study. **I**. Biosynthesis of fatty acids after 24 h under standard conditions. **II**. Biosynthesis of fatty acids after 48 h under standard conditions. **III**. Biosynthesis of fatty acids after 24 h under oxidative stress conditions. **IV.** Biosynthesis of fatty acids after 48 h under oxidative stress conditions.

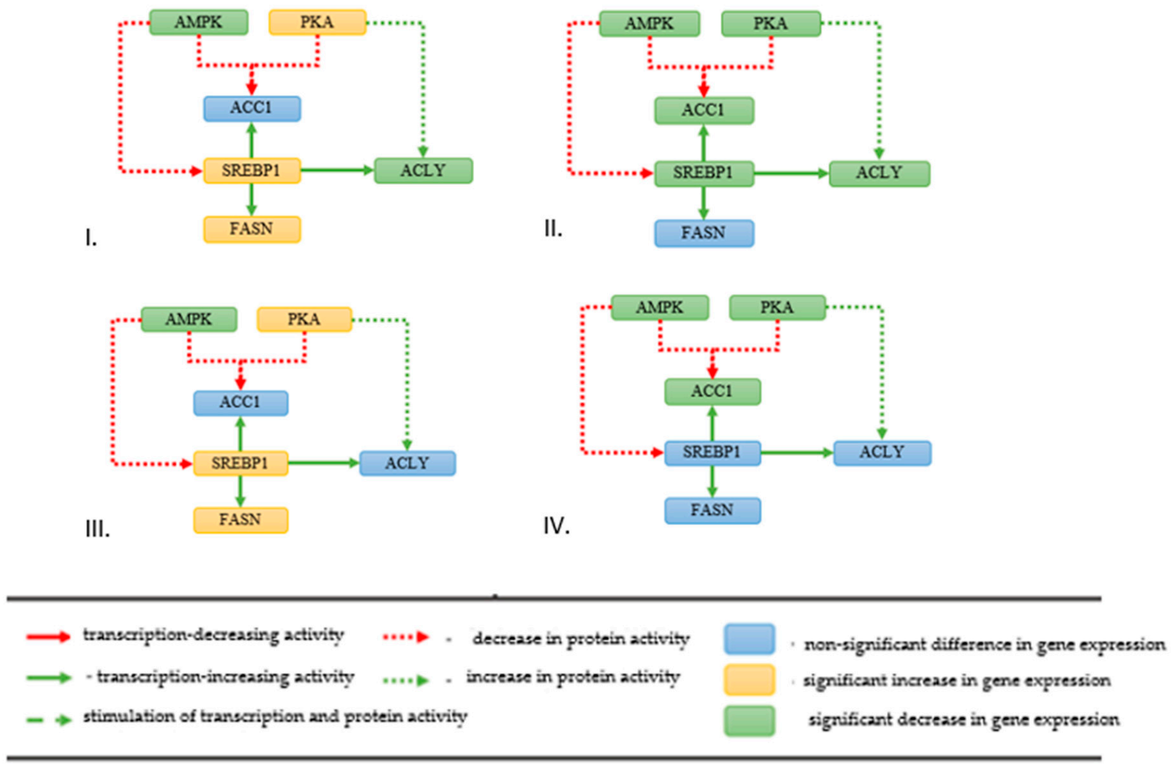

Figure S13. Interactions related to fatty acid metabolism in 3T3-L1 adipocytes treated with a mixture of a mixture of resveratrol (75 µM) and *trans-10, cis-12* CLA (50 µM) under standard and oxidative stress conditions, based on the scientific literature presented and the results obtained in this study. **I**. Biosynthesis of fatty acids after 24 h under standard conditions. **II**. Biosynthesis of fatty acids after 48 h under standard conditions. **III**. Biosynthesis of fatty acids after 24 h under oxidative stress conditions. **IV.** Biosynthesis of fatty acids after 48 h under oxidative stress conditions.

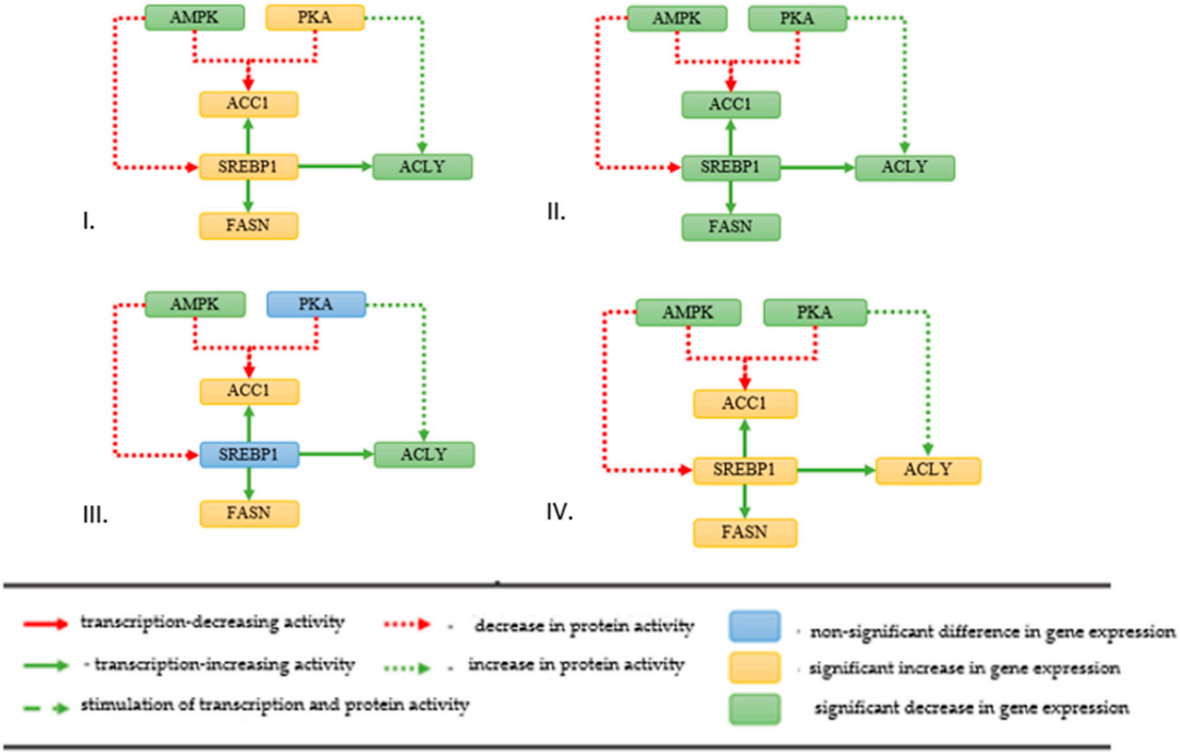

Figure S14. Interactions related to fatty acid metabolism in 3T3-L1 adipocytes treated with an equimolar mixture of resveratrol, *cis-9, trans-11* CLA and *trans-10, cis-12* CLA under standard and oxidative stress conditions, based on the scientific literature presented and the results obtained in this study. **I**. Biosynthesis of fatty acids after 24 h under standard conditions. **II**. Biosynthesis of fatty acids after 48 h under standard conditions. **III**. Biosynthesis of fatty acids after 24 h under oxidative stress conditions. **IV.** Biosynthesis of fatty acids after 48 h under oxidative stress conditions.
